# Denoising perturbation signatures reveal an actionable AKT-signaling gene module underlying a poor clinical outcome in endocrine-treated ER+ breast cancer

**DOI:** 10.1186/s13059-015-0630-4

**Published:** 2015-04-02

**Authors:** Andrew E Teschendorff, Linlin Li, Zhen Yang

**Affiliations:** CAS Key Laboratory of Computational Biology, Chinese Academy of Sciences-Max Planck Partner Institute for Computational Biology, Shanghai Institutes for Biological Sciences, Chinese Academy of Sciences, 320 Yue Yang Road, Shanghai, 200031 China; Statistical Cancer Genomics, Paul O’Gorman Building, UCL Cancer Institute, University College London, 72 Huntley Street, London, WC1E 6BT UK

## Abstract

**Background:**

Databases of perturbation gene expression signatures and drug sensitivity provide a powerful framework to develop personalized medicine approaches, by helping to identify actionable genomic markers and subgroups of patients who may benefit from targeted treatments.

**Results:**

Here we use a perturbation expression signature database encompassing perturbations of over 90 cancer genes, in combination with a large breast cancer expression dataset and a novel statistical denoising algorithm, to help discern cancer perturbations driving most of the variation in breast cancer gene expression. Clustering estrogen receptor positive cancers over the perturbation activity scores recapitulates known luminal subtypes. Analysis of individual activity scores enables identification of a novel cancer subtype, defined by a 31-gene AKT-signaling module. Specifically, we show that activation of this module correlates with a poor prognosis in over 900 endocrine-treated breast cancers, a result we validate in two independent cohorts. Importantly, breast cancer cell lines with high activity of the module respond preferentially to PI3K/AKT/mTOR inhibitors, a result we also validate in two independent datasets. We find that at least 34 *%* of the downregulated AKT module genes are either mediators of apoptosis or have tumor suppressor functions.

**Conclusions:**

The statistical framework advocated here could be used to identify gene modules that correlate with prognosis and sensitivity to alternative treatments. We propose a randomized clinical trial to test whether the 31-gene AKT module could be used to identify estrogen receptor positive breast cancer patients who may benefit from therapy targeting the PI3K/AKT/mTOR signaling axis.

**Electronic supplementary material:**

The online version of this article (doi:10.1186/s13059-015-0630-4) contains supplementary material, which is available to authorized users.

## Background

Tumors are often found to carry a large number of aberrations, including genetic mutations, genomic copy-number aberrations, as well as epigenetic changes [1-3]. Irrespective of the underlying mechanism, if the resulting changes are functional, then these may cause downstream changes in signaling pathway activity resulting in abnormal cellular features such as uncontrolled cell growth or evasion of apoptosis. However, it is thought that only a relatively small fraction of the observed aberrations, even if functional, constitute important drivers of tumor growth and progression [4,5].

Although recent The Cancer Genome Atlas (TCGA) studies have identified many candidate driver mutations and copy-number aberrations across different cancer types, the net effect of such perturbations in any given cancer might be hard to predict [6,7]. Indeed, as pointed out recently by Gatza et al. [8], the mere presence of a candidate driver mutation in a given cancer does not imply that the associated signaling pathway is necessarily deregulated. Thus, to realize the goals of personalized medicine, one needs to assess the functional consequence of specific cancer perturbations in the cancer of a given patient. This in turn requires the analysis of functional data, for instance gene or protein expression/activity. As advocated here, and also in Gatza et al. [8], one way to address this formidable challenge is to assess the *in vivo* activity of cancer perturbations by interrogating prior, possibly *in vitro* derived, perturbation gene expression signatures in the transcriptomic profile of the given cancer. In our context, a perturbation experiment describes the effect on the cellular phenotype of a functional change to a single (or a few) gene(s) [6]. This perturbation approach may not only help dissect driver and passenger events, but also help define patient subgroups who might benefit from specific targeted drug treatments [6,9].

However, to use perturbation gene expression signatures to estimate perturbation or pathway activity scores in tumors is a complex task. Indeed, we have argued in the past that naive computation of these activity scores may result in highly suboptimal inferences, because many of the genes making up perturbation signatures may reflect confounding sources of variation, and thus represent false positives [10,11]. One immediate reason why this may be so, is that single perturbation experiments can only be studied properly in an *in vitro* setting, which inevitably ignores the effects of the tumor microenvironment [12,13]. Thus, translating the effects of gene perturbations in cell-line models to primary tumour samples is a complex endeavor due to the effects of the tumor microenvironment, but also due to variations in the biological background (no given cell line can recapitulate the precise aberration profile of an *in vivo* tumor sample) and complex *in vitro* effects. As a result of this, we have argued that such perturbation signatures must be *denoised* before using them to estimate perturbation activity scores in individual tumor samples or cancer cell lines [11]. To this end, we developed a statistical algorithm, called DART (Denoising Algorithm using Relevance network Topology), which allows a denoising of the perturbation signature in the data of interest to be performed [11]. Underlying this DART methodology is the hypothesis that a subset of the genes making up the perturbation signatures may indeed be relevant in the cancer of interest [11]. DART allows this hypothesis to be tested by assessing the consistency of the gene expression correlation patterns in relation to those predicted by the prior information from the *in vitro* signature. Importantly, we showed that the filtering and denoising step implemented in DART, improved statistical inference of perturbation/pathway activity levels [11].

Here, we further improve on the existing DART algorithm, and apply the improved method to the problem of endocrine resistance in estrogen receptor positive (ER+) breast cancer [14,15]. It is well known that a significant proportion of ER+ breast cancers do not respond well to endocrine therapy, and for which there is still a lack of alternative therapeutic targets. Thus, our aim is to use a novel perturbation gene expression signature approach to identify subgroups of ER+ patients who respond less well to endocrine therapy, but who may benefit from treatments targeting cancer genes that are active in these tumor subgroups. To address this, we integrate a large database of over 90 gene expression perturbation signatures, reflecting perturbations of many important breast cancer genes, with the most comprehensive breast cancer gene expression dataset, the METABRIC set, comprising two independent subsets of 774 (the discovery set) and 651 (the validation set) ER+ samples, respectively [3]. In performing this integration, we first identify the perturbations that drive most of the variation in gene expression across breast cancer. Subsequently, we explore the molecular taxonomy of ER+ breast cancer, which results from analyzing the activity patterns of these perturbation signatures. Focusing on those activity profiles representing actionable perturbations and predicting endocrine resistance in ER+ breast cancer, we identify a 31-gene AKT signaling module, which also predicts sensitivity to AKT/PI3K/mTOR inhibitors [16,17], thus providing a means of identifying ER+ patients who could potentially benefit from such treatment.

## Results

### Variable perturbation signatures in breast cancer

**Figure 1:**
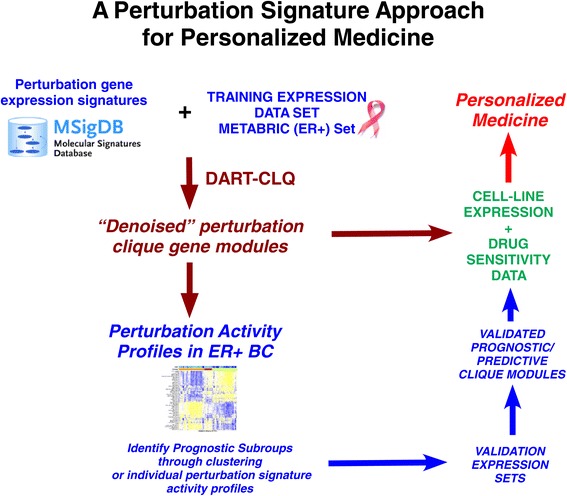
Overall strategy. A database of (*in vitro*) perturbation gene expression signatures is denoised for relevance in a particular cancer type (here ER+ breast cancer) by using a large training expression set of (*in vivo*) tumor samples, representative of that cancer type. The denoising is done with the DART-CLQ algorithm (see Figure 2), which results in a small number of clique gene modules, from which improved estimates of perturbation activity can be derived. Using the perturbation activity score matrix one can then identify associations between perturbations and clinical outcome. The same clique gene modules allow estimation of perturbation activity scores in independent *in vivo* tumor sample datasets and in panels of cell lines, allowing associations with outcome to be validated, and to identify potential drug treatments that may benefit certain patient subgroups. BC, breast cancer; ER+, estrogen receptor positive; MSigDB, Molecular Signatures Database.

**Figure 2:**
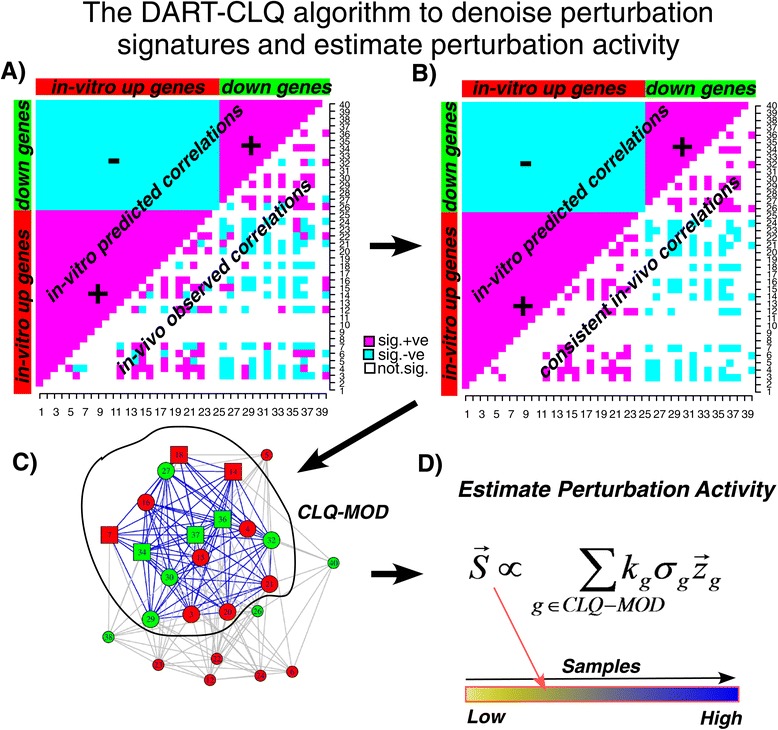
The DART-CLQ algorithm.**(A)** Given an *in vitro* perturbation signature (depicted one of 40 genes) of up- and downregulated genes, one computes gene pairwise correlations in expression over the samples of a large training set. One would expect two genes that are commonly up- or downregulated in the *in vitro* signature to exhibit positive correlations, whereas two genes with one up- and the other downregulated, would be predicted to be anti-correlated (upper diagonal). If the perturbation signature has explanatory power in the training expression set, one would expect that observed correlations (lower diagonal) should agree, statistically, with the predicted ones, and if so, a consistency score can be derived. **(B)** If the consistency score is statistically significant, the correlation network is pruned to remove those observed correlations for which the directionality is inconsistent with the prior information, leaving only consistent and significant correlations. **(C)** The consistent and significant correlations define a correlation network with a maximally connected component as depicted. DART-CLQ infers all the largest cliques in this component and merges them (in practice, largest cliques exhibit very strong overlaps) to define an approximate clique gene module (CLQ-MOD). An example of a clique within this module is indicated by the square nodes (genes). **(D)** Given the approximate clique gene module, perturbation activity is now estimated by first z-normalizing each of a module gene’s expression profile (mean centering and unit variance scaling) over the samples and then constructing a weighted average, where we weight each gene according to its degree in the module (*k*_g_) and whether it was predicted to be up- (*σ*_g_=1) or downregulated (*σ*_g_=−1). We note that the difference between DART-CLQ and DART is that DART estimates the activity score over the full maximally connected correlation network.

The overall strategy we propose is summarized in Figure 1. We first assembled a database of 90 perturbation expression signatures from the C6 signature class of the Molecular Signatures Database (MSigDB) (Figure 1, Materials and methods). The perturbations included activation (e.g., using a retrovirus to overexpress an oncogene) or silencing (e.g., an RNA interference experiment), and targeted many genes that are important in cancer, including breast cancer (e.g., *ERBB2*, *TP53*, *MYC*, *AKT*, *RB1*, *CCND1*, etc., see Additional file 1: Table S1). Each one of these perturbation signatures consisted of two gene lists, one involving genes upregulated in response to the perturbation, and another involving genes that are downregulated. Since these perturbations were artificially induced in cell-line models, we wanted to first assess if the patterns of up- and downregulation of the genes making up these perturbation signatures are reflected in their correlative expression patterns across primary breast tumors (Figures 1 and 2, Materials and methods) [11]. To this end, we first used the normalized gene expression data from the METABRIC discovery cohort [3], encompassing almost 1,000 different breast cancers to estimate gene pairwise correlations in expression for all the genes within a given perturbation signature (Figures 1 and 2, Materials and methods). The overall scheme for an example perturbation signature is shown in Figure 2. For each signature, this resulted in a heat map of statistically significant gene pairwise correlations (Figure 2A). To assess consistency of the correlative patterns with the predictions from the perturbation signature, we count the number of pairs where the directionality is consistent with the prior *in vitro* information (Figure 2A, Materials and methods). Statistical significance of this consistency score is obtained by Monte Carlo simulation as described by us previously [11]. Thus, this procedure results in a consistency and significance score for each perturbation signature, which tells us whether this signature exhibits patterns of variation in the METABRIC set which is consistent with the patterns of up- and downregulation predicted by the *in vitro* experiment. We performed this consistency analysis separately for the ER+ and ER − breast cancer METABRIC subsets, since ER controls the expression of a very large number of genes, which could confound correlation and skew statistical significance estimates.

Of the 90 perturbation signatures, we found that only 57 and 38 induced correlation networks in the ER+ and ER − subsets, respectively, which were significantly consistent with the prior *in vitro* predictions (Monte Carlo test *P*<0.001, Materials and methods). Thus, in the ER − case, less than 50 *%* of the *in vitro* perturbation signatures showed correlative patterns consistent with the prior information. Only for the perturbation signatures deemed consistent by this analysis, can we assume that the inter-tumor expression variability of the genes making up these signatures reflects corresponding inter-tumor variations in the activity of the given perturbation. Hence, perturbation activity profiles are only computed for these significantly variable and consistent signatures.

### The DART-CLQ algorithm and validation of perturbation activity estimates

To compute perturbation activity values for one of these consistent signatures, we first prune the correlation network by removing those pairs (edges) where the directionality of correlation was inconsistent with the prior information (Figure 2B). This results in a pruned correlation network with a large maximally connected component, and with isolated nodes representing genes in the signature that do not show any significant or consistent correlations (and which are hence removed from the process). In our previous DART algorithm, perturbation activity would be estimated over the whole maximally connected network [11]. However, this often resulted in averaging expression profiles over a relatively large number of genes. We posited that improved inference could be obtained by focusing on the largest cliques in the pruned correlation network. Although the largest cliques may not be unique, we found that they generally exhibited very strong overlaps, justifying their merging, and resulting in approximate clique modules typically of size of ∼10 to 100 genes (Figure 2C). The resulting novel algorithm, called DART-CLQ (denoising algorithm using relevance network topology and cliques), thus estimates perturbation activity by constructing a weighted average of normalized expression profiles over the genes in this approximate clique gene module (Figure 2D).

**Figure 3:**
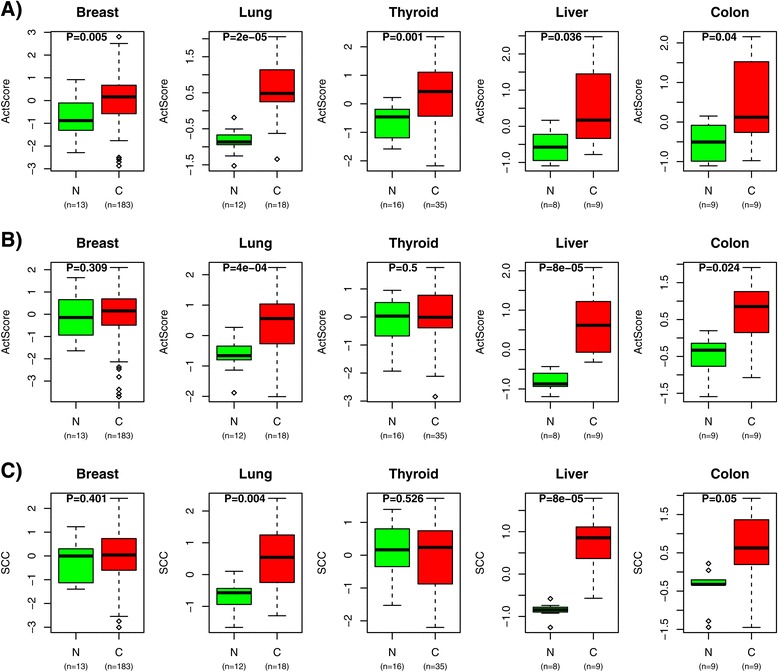
Validation of the DART-CLQ algorithm.**(A)** Box plots of predicted activity scores from DART-CLQ of a p53 deactivation signature between normal (N) and cancer (C) tissue in five different tissue types from the Affymetrix study of Yu et al. [18]. Number of samples being compared are given below each box plot. *P* value is from a Wilcoxon rank sum test. **(B)** As (A), but for activity scores estimated using DART. **(C)** As (A), but for activity scores estimated using the Spearman correlation coefficient (SCC). C, cancer; N, normal; SCC, Spearman correlation coefficient.

To validate DART-CLQ, we first focused on a well-known cancer gene, *TP53*. An associated perturbation signature, reflecting *TP53* deactivation, was also among the consistent signatures derived in the ER+ subset of breast cancer, resulting in a 35-gene clique module. Although *TP53* is not mutated in all cancers, deactivation of *TP53* signaling is a near-universal feature of cancer, and so we argued that the best possible test of DART-CLQ would be a comparison of *TP53* activity levels between normal and cancer tissue. Indeed, we posited that the *TP53* clique module, although derived from the perturbation signature in ER+ breast cancer, would exhibit a higher signature score in cancer tissue compared to normal samples: we note that since the original *in vitro* perturbation signature measures deactivation, that a higher signature score in cancer is consistent with a higher frequency of inactivation in the neoplasias. To test this, we computed activity scores for the *TP53* DART-CLQ module in an Affymetrix gene expression dataset encompassing normal and cancer samples for five different tissue types [18], for which there was also associated RNA sequencing (RNA-seq) expression data from the TCGA (Materials and methods). This confirmed that the score was indeed higher in cancer independent of tissue type (Figure 3A). Importantly, using our original DART algorithm, or using naive Spearman rank correlations to derive the score did not result in significant differences across all cancer types (Figure 3B,C, Materials and methods), supporting the view that DART-CLQ achieves more reliable activity estimates. The improved robustness of DART-CLQ over DART and the Spearman correlation was further confirmed in the same tissue types using five independent normal/cancer RNA-seq expression sets from the TCGA (Additional file 1: Figure S1).

To test DART-CLQ further, we observed that a number of independently derived perturbation signatures, but which affected the same oncogenes and tumor suppressors, generated perturbation activity profiles that were highly correlated, indicating the consistency and integrity of the procedure. For example, two different perturbation signatures of *STK33* yielded cliques of 38 and 44 genes, respectively, with a common overlap of only 14 genes, yet their activity profiles were highly correlated (Additional file 1: Figure S2). Similarly, two perturbation signatures for *KRAS* activation, derived for two different tissue types, also revealed broad statistical agreement (Additional file 1: Figure S2) despite there being no gene in common between the two clique modules of 16 and 30 genes, respectively. Using our previous DART algorithm, or naive Spearman rank correlations, to derive the activity scores resulted in lower *R*^2^ values for *STK33* and in non-significant positive correlations for *KRAS* (Additional file 1: Figure S2), once again supporting the view that DART-CLQ achieves more reliable activity estimates.

### Perturbation signature activity profiles recapitulate known breast cancer subtypes

**Figure 4:**
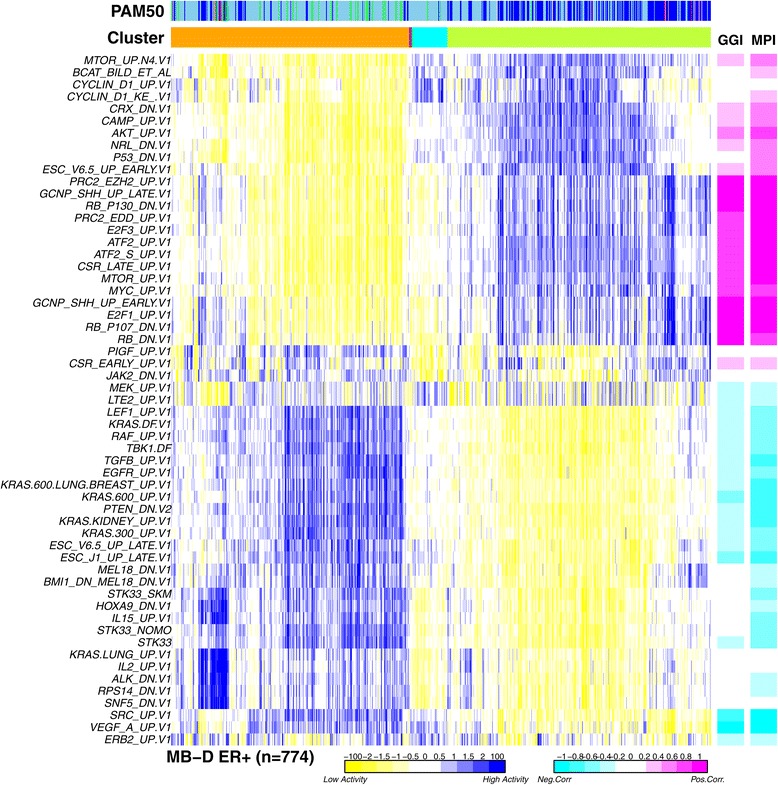
Perturbation signature activity scores recapitulate known subtypes of ER+ breast cancer. ConsensusCluster heat map of perturbation activity scores across the 774 ER+ breast cancers of the METABRIC discovery set. Samples clustered using the ConsensusCluster algorithm (the five-cluster solution is shown). Distribution of breast cancer intrinsic subtypes (PAM50) across the five inferred clusters is indicated (sky blue: luminal-A, blue: luminal-B, green: normal-like, pink: HER2+, red: basal). Perturbation signatures have been clustered using hierarchical clustering (dendrogram has been suppressed) according to similarity of activity profiles over samples. Color bars to the right depict the Pearson correlations of the perturbation activity scores with the GGI and MPI. ER+, estrogen receptor positive; GGI, genome grade index; MB-D, METABRIC discovery set; MPI, molecular prognostic index.

Having demonstrated that DART-CLQ obtains sensible activity estimates and having identified the most variable and consistent perturbation signatures, we next asked how ER+ breast cancer samples would cluster over the estimated perturbation activity profiles. Applying ConsensusCluster [19], we identified a near optimal five-cluster solution, with two main clusters of 341 and 377 samples, respectively, one intermediate-sized cluster (51 samples) and two clusters consisting of a few outliers (4 and 1 samples, respectively) (Figure 4). Demonstrating the biological significance of the activity patterns, the two main clusters correlated strongly with luminal A/B subtype status (Fisher test, *P*<2×10^−16^). Indeed, many of the perturbation signatures correlated with luminal A/B subtype status (Additional file 1: Table S2). For instance, luminal-B tumors were characterized by high activity of specific activating perturbations, such as high polycomb *EZH2*, *EDD* and high *SHH*, *E2F3*, *MYC* signaling Figure 4 and Additional file 1: Table S2). Luminal-B tumors were also characterized by high activity of deactivation signatures such as that of well-known tumor suppressors (e.g., *RB* and *P53*), but also that of less well-known genes such as *CRX* and *NRL* (Figure 4). In general, those perturbation signatures that were highly active in the luminal-B enriched cluster also exhibited strong positive correlations with proliferation prognostic indices such as the genome grade index (GGI) [20] or the molecular prognostic index (MPI) [21] (Figure 4). In particular, this was the case for the *RB* deactivation and *E2F1/E2F3* activation signatures, which are known to regulate cell proliferation directly (Figure 4). We further note that we observed strong positive correlations between MYC and E2F3 activation signatures (Pearson ∼0.75) consistent with E2F3 being a downstream target of MYC. An even stronger correlation was observed between the RB deactivation and E2F3 activation signature (Pearson >0.85), also consistent with RB being a direct repressor of E2F3. Luminal-A tumors were characterized by high activity of *EGFR*, *RAF*, *KRAS* activating signatures and surprisingly by a *PTEN* deactivation signature. Of note, the smaller intermediate cluster defined another luminal-A subtype, characterized by high activity of *CCND1*, beta-catenin and MTOR signaling.

### An AKT gene module correlates with poor outcome in endocrine-treated ER+ breast cancer

Next, we decided to focus on the specific subset of ER+ patients who only received anti-hormone therapy (tamoxifen or aromatase inhibitors) with or without radiotherapy. It is well known that not all ER+ patients respond well to tamoxifen, yet the molecular basis for this is still unclear [14,15], and there is currently also a lack of alternative therapies for this subgroup of patients. Thus, to identify potential alternative treatments (Figure 1), we first carried out a survival analysis for the 57 consistent and highly variable perturbation signatures, focusing on endocrine-treated ER+ patients only. To gain power, we merged the two METABRIC cohorts, resulting in 926 eligible patients, and used independent datasets for validation. Of the 57 perturbation signatures, 42 were significantly associated with clinical outcome in univariate analysis (Additional file 1: Table S3 and Figure S3), with 17 of these also remaining significant in multivariate analysis adjusted for stage, grade and tumor size, and after correction for multiple testing (Benjamini–Hochberg false discovery rate, FDR <0.15, Table 1).

**Table 1:** **Perturbation signature modules from DART-CLQ predicting outcome in the endocrine-treated ER+ METABRIC set in both univariate and multivariate analysis (adjusted for grade, stage and tumor size)**

	**Univariate**		**Multivariate**		
	***n*** **=926**		***n*** **=685**		
**Module**	**HR (95% CI)**	***P*** *	**HR (95% CI)**	***P*** *	**Class**
CYCLIN_D1_KE(UP)	1.14 (1.04–1.25)	0.005	1.12 (1.01–1.25)	0.039	Prolif
CSR_LATE_UP	1.19 (1.08–1.32)	0.001	1.13 (1.01–1.27)	0.039	NotTgt
AKT_UP	1.18 (1.07–1.31)	0.001	1.13 (1.01–1.27)	0.041	**Tgt**
PIGF_UP	0.86 (0.78–0.94)	0.001	0.88 (0.79–0.97)	0.014	HR <1
ATF2_S_UP	1.2 (1.08–1.32)	0.001	1.14 (1.01–1.28)	0.035	NotTgt
ATF2_UP	1.21 (1.09–1.34)	<0.001	1.15 (1.02–1.3)	0.019	NotTgt
E2F3_UP	1.21 (1.1–1.33)	<0.001	1.13 (1.01–1.27)	0.033	Prolif
SRC_UP	0.83 (0.75–0.91)	<0.001	0.88 (0.78–0.99)	0.036	HR <1
PRC2_EDD_UP	1.21 (1.1–1.33)	<0.001	1.15 (1.02–1.29)	0.018	NotTgt
MTOR_UP.N4	1.19 (1.08–1.32)	0.001	1.15 (1.02–1.3)	0.022	**Tgt**
PTEN_DN.V2	0.84 (0.76–0.94)	0.001	0.87 (0.76–0.98)	0.023	NotTgt
RB_P107_DN	1.2 (1.09–1.31)	<0.001	1.13 (1.01–1.27)	0.037	NotTgt
RB_P130_DN	1.21 (1.1–1.34)	<0.001	1.13 (1–1.27)	0.044	NotTgt
KRAS.300_UP	0.78 (0.7–0.87)	<0.001	0.79 (0.7–0.89)	<0.001	NotTgt
KRAS.600_UP	0.82 (0.74–0.92)	<0.001	0.83 (0.73–0.94)	0.004	NotTgt
KRAS.600.LUNG.BREAST_UP	0.84 (0.75–0.94)	0.003	0.84 (0.74–0.96)	0.01	NotTgt
KRAS.KIDNEY_UP	0.83 (0.75–0.92)	<0.001	0.85 (0.76–0.96)	0.008	NotTgt

Hazard ratios (HRs), 95 *%* confidence intervals (CIs) and Cox regression *P* values are indicated. Perturbation activity scores were scaled to unit variance before running the Cox regression to allow meaningful comparison of HR values. Last column labels the original perturbation signatures according to whether they are targetable (Tgt, i.e., they exhibit HR >1 and there are drug inhibitors for them), not targetable (NotTgt, i.e., either no specific drug exists, or a drug does exist but the module exhibits HR <1), or if they are directly implicated in the cell cycle/proliferation (Prolif). *All *P* values reported in this table pass a Benjamini–Hochberg corrected FDR threshold of 0.15.

Given that random gene expression signatures have often been shown to correlate spuriously with outcome [22,23], it is important to assess the overall prognostic significance of the 17 associations in Table 1 by another means. As a first test, we constructed a set of 90 random perturbation signatures, matched for the same size and distribution of up- and downregulated genes as the original 90 MSigDB perturbation signatures (Materials and methods). These random signatures were run through the same DART-CLQ algorithm on the ER+ METABRIC dataset. Because the signatures are random, we would not expect their consistency scores to be statistically significant. Confirming this, none of the 90 random signatures attained consistency scores that passed the significance level of 0.001, which contrasts strongly with the 57 MSigDB perturbation signatures that did pass this level of significance (Additional file 1: Figure S4). Next, we decided to test expression signatures from an unrelated biological context, following the strategy of Venet et al. [22]. Specifically, we used a large expression dataset of 353 normal tissue specimens from 65 different anatomical sites [24], to derive signatures of differential expression between tissue types that are unrelated to breast, including brain, lymph nodes and prostate (Materials and methods). As in Venet et al., we reasoned that these tissue-specific signatures should not exhibit as strong prognostic association as our 17 DART-CLQ modules, if the latter represent genuine associations. Applying DART-CLQ to a total of 66 different signatures derived from pairwise comparisons of 12 anatomical sites, revealed that 63 of these had a significant consistency score P-value (*P*<0.001). For these 63 signatures, we then performed Cox regressions in the endocrine-treated ER+ METABRIC samples, both univariately and multivariately. Next, we counted the number of signatures with a Cox *P* value more extreme than the largest Cox *P* value reported in Table 1. In the univariate case, we observed 12 signatures, i.e., 19 *%* of the 63 signatures, with a more extreme *P* value than 0.005 (FDR <0.15), and only 8 (i.e., 12 *%*) with a *P* value less than 0.002 (Additional file 1: Figure S5). A similar result was obtained in the multivariate case (Additional file 1: Figure S5). Thus, we find a good agreement between our earlier FDR estimate of 0.15 and these estimates obtained by picking signatures that should be unrelated to breast cancer. In summary, this randomization analysis supports the view that the prognostic associations in Table 1 are genuine and not due to random chance.

Of the 17 prognostic DART-CLQ modules, those correlating most strongly with outcome corresponded to KRAS perturbations (a total of four signatures, Table 1). KRAS, however, is notoriously difficult to target. Another four of the top signatures (e.g., two RB deactivation, one E2F3 and one CCND1 signature) were directly related to proliferation (Table 1 and Figure 4). Of the remaining nine signatures, only four are clearly targetable (PIGF, SRC, AKT(AKT1) and mTOR). However, only for the AKT and mTOR modules did we observe that high activity predicted poor clinical outcome (HR >1, Table 1). Although AKT and mTOR signaling are closely related signaling pathways, we decided to focus on the AKT module, because the original AKT perturbation signature was derived by explicit overexpression of *AKT1*, in contrast to the mTOR signature that had been derived by the action of everolimus [25]. Moreover, aberrant AKT signaling has been proposed as one potential mechanism underlying endocrine resistance in ER+ breast cancer [15]. On the other hand, recent work has also shown that activating mutations in *PIK3CA*, a frequent alteration in breast cancer [3], does not predict the response to endocrine treatment [26-28]. We therefore decided to explore if a functional marker of aberrant AKT signaling (such as our AKT gene module) may be a more relevant indicator of endocrine resistance.

**Figure 5:**
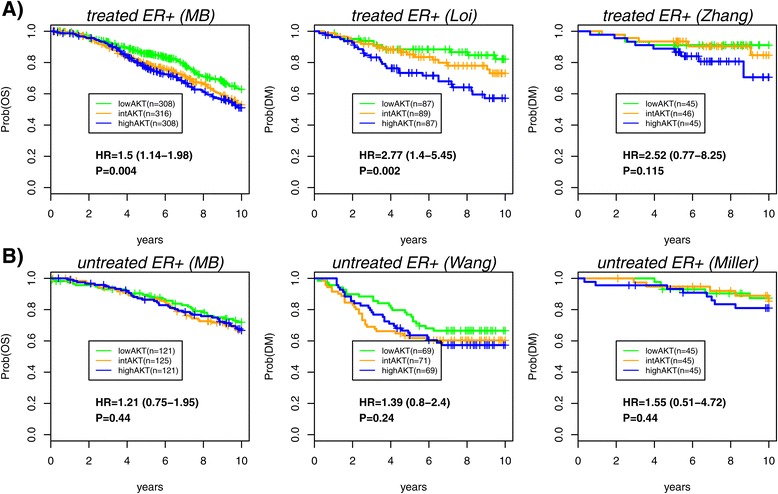
Kaplan–Meier survival analysis of the AKT gene module in ER+ breast cancer.**(A)** Kaplan–Meier curves of the endocrine-treated ER+ patients in the METABRIC set as well as for the datasets of Loi et al. [20] and Zhang et al. [30], with samples stratified according to the lower, intermediate and upper tertiles of the AKT module activation score. Number of samples in each tertile is indicated. Hazard ratios, 95 *%* confidence intervals and Cox regression *P* values between lower and higher tertiles are given. **(B)** As (A) but for the untreated ER+ patients of the METABRIC set, as well as the untreated ER+ patients of Wang et al. [29] and Miller et al. [31]. Untreated here means no endocrine therapy or chemotherapy, but may include radiotherapy. ER+, estrogen receptor positive; HR, hazard ratio; MB, METABRIC; Prob, probability; OS, overall survival; DM, distant metastasis.

To explore this, we first assessed whether the AKT module correlates with outcome in untreated (no chemotherapy or endocrine therapy but may include radiotherapy) ER+ patients. In contrast with the endocrine-treated subset, the association in the untreated group was no longer significant (Table 2). To validate the results, we collected another two large ER+ gene expression datasets, matched for sample size, with everyone in one cohort having received tamoxifen [20], whilst in the other the patients were all untreated (i.e., they received only radiotherapy) [29]. This analysis confirmed that the association with outcome was specific to the treated ER+ cohort (Table 2). To validate the findings further, we collected yet another pair of ER+ datasets, albeit of smaller sample size, one treated with tamoxifen [30] whereas the other was untreated (i.e., they received only radiotherapy) [31]. Once again, we found that the AKT module was more strongly associated with outcome for the ER+ endocrine-treated cohort (Table 2). A separate meta-analysis for the three treated and three untreated ER+ cohorts further confirmed the strong statistical significance of the association in the endocrine-treated cohorts, with no significance in the untreated sets (Table 2). The ability of the AKT gene module to stratify treated ER+ patients with different survival rates was confirmed further by Kaplan–Meier analysis (Figure 5).

**Table 2:** **Cox regression results of the AKT gene module**

**Treated ER+ cohort**	**METABRIC**	**Loi**	**Zhang**	**Combined Fisher test**
Univariate	*n*=926	*n*=250	*n*=136	
HR (95*%*)	1.18 (1.07–1.31)	1.6 (1.24–2.05)	1.56 (0.97–2.51)	
*P*	0.001	0.0003	0.06	<0.00001
Multivariate	*n*=685	*n*=207	*n*=136^a^	
HR (95*%*)	1.13 (1.00–1.27)	1.49 (1.11–2.00)	1.56 (0.97–2.51)	
*P*	0.041	0.008	0.06	0.001
**Untreated ER+ cohort**	**METABRIC**	**Wang**	**Miller**	**Combined Fisher test**
Univariate	*n*=360	*n*=209	*n*=127	
HR (95*%*)	1.12 (0.95–1.32)	1.13 (0.91–1.39)	1.32 (0.83–2.10)	
*P*	0.17	0.26	0.23	0.16
Multivariate	*n*=272	*n*=209^a^	*n*=126	
HR (95*%*)	1.09 (0.89–1.34)	1.13 (0.91–1.39)	0.96 (0.61–1.54)	
*P*	0.41	0.26	0.88	0.58

These are for endocrine-treated [20,30] and untreated [29,31] ER+ cohorts, as indicated, as well as for a meta-analysis under a combined Fisher test. Untreated means cases did not receive either endocrine or chemotherapy, but may have received radiotherapy. The number of samples, hazard ratios (HRs), 95 *%* confidence intervals and log-rank test *P* values are given for both univariate and multivariate (adjusted for stage/nodal status, grade and tumor size) analyses. Perturbation activity scores were scaled to unit variance before running the Cox regressions to allow meaningful comparison of HR values.

^a^Note that for the Wang dataset [29], all samples were lymph node negative and no information on tumor size and grade was available. Similarly, for the Zhang dataset [30], no additional clinical information was available. For the Miller dataset [31], the multivariate analysis was adjusted for size and grade only since all untreated samples were lymph node negative. For the METABRIC set, overall survival was used as the endpoint; for all others we used distant-metastasis-free survival. In all cases, outcome data were censored at 10 years.

### The AKT gene module predicts sensitivity specifically to AKT/mTOR drug inhibitors

**Figure 6:**
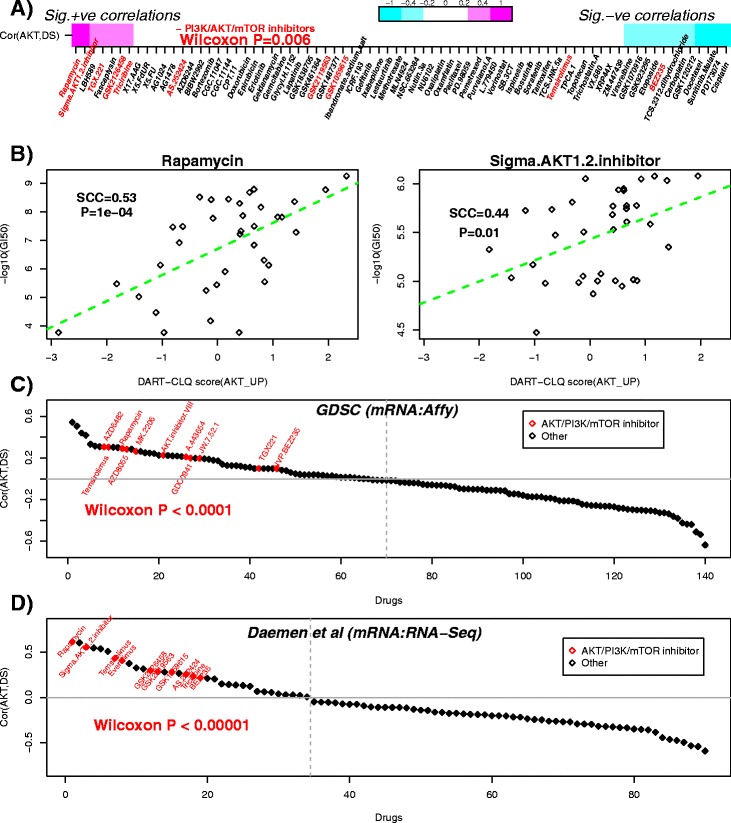
AKT gene module predicts sensitivity to AKT/mTOR signaling inhibitors.**(A)** Spearman rank correlation between the predicted AKT perturbation activity score and drug sensitivity (− log10GI50) values assessed over a panel of 45 breast cancer cell lines. Drugs have been ranked according to strength of correlation. Magenta: significant positive correlation, white: no significant correlation, cyan: significant negative correlation. Drugs that are PI3K/AKT/mTOR inhibitors are indicated in red. The Wilcoxon rank sum test *P* value testing for any skew of these inhibitors towards positive correlations is given. **(B)** Scatter plots of drug sensitivity against AKT activity scores for the two top ranked drugs in (A). Spearman rank correlation coefficients (SCCs) and associated *P* values are indicated. **(C)** Spearman rank correlation between the predicted AKT perturbation activity score and drug sensitivity (− log10GI50) values as assessed over a panel of 39 breast cancer cell lines from the Genomics and Drug Sensitivity in Cancer project. Note that for this set, the number of cell lines for each drug differs, hence individual *P* values are suppressed. Drugs that are PI3K/AKT/mTOR inhibitors are indicated in red. The Wilcoxon rank sum test *P* value testing for a positive skew of the PI3K/AKT/mTOR inhibitors towards positive correlations is given. **(D)** As (C), but for the data from Daemen et al. [34], which used − log10GI50 values. Correlations estimated over 42 breast cancer cell lines. Drugs that are PI3K/AKT/mTOR inhibitors are indicated in red. The Wilcoxon rank sum test *P* value testing for a positive skew of the PI3K/AKT/mTOR inhibitors towards positive correlations is given. +ve, positive; −ve, negative; Cor, correlation; GDSC, Genomics Drug Sensitivity in Cancer Project; RNA-seq, RNA sequencing; SCC, Spearman rank correlation coefficient; Sig., significant; DS, drug sensitivity.

Since several AKT/mTOR pathway inhibitors have been shown to be effective for endocrine-resistant breast cancer cell lines [32], we decided next to investigate if the *in vivo* derived AKT gene module would be able to predict the response to drugs targeting the AKT/mTOR pathway. We collected an expression dataset of 45 breast cancer cell lines, for which a drug sensitivity screen, with − log10GI50 scores, for over 70 drugs had also been performed [16], including known AKT inhibitors (Sigma1.2 and triciribine), PI3K inhibitors (TGX221, GSK2119563, GSK1059615 and AS.252424), mTOR inhibitors (rapamycin and temsirolimus) and dual PI3K/mTOR inhibitors (GSK2126458 and BEZ235). Using the same AKT clique module inferred for the METABRIC discovery set, we estimated perturbation activity scores for the breast cancer cell-line panel. The obtained activity scores should reflect the relative activity of AKT signaling in the breast cancer cell lines, and thus we posited that the drug sensitivity values correlating most strongly with the activity scores would be those of PI3K/AKT/mTOR inhibitors. Confirming this, the top seven correlated drugs included five PI3K/AKT1/mTOR inhibitors, i.e., rapamycin, Sigma1.2, TGX221, GSK2126458 and triciribine (Figure 6A). We note that although the *P* values were only significant for rapamycin and Sigma1.2, the probability of observing so many PI3K/AKT/mTOR inhibitors among the top-ranked drugs, purely by random chance, is only 0.006 (one-sided Wilcoxon rank sum test, Figure 6A,B). Interestingly, had we used the original full AKT perturbation signature and a Spearman correlation (i.e., without using DART-CLQ) to estimate perturbation activity, we would have found that the top drug (gefitinib) is not a PI3K/AKT/mTOR inhibitor, with rapamycin and Sigma1.2 only ranking fourth and tenth, respectively. Moreover, we would have observed tamoxifen to be ranked higher than Sigma1.2, whereas using DART-CLQ, tamoxifen was ranked much lower, as we would expect from a gene module that predicts insensitivity or resistance to tamoxifen (Figure 6A). Using DART-CLQ, we also observed that breast cancer cell lines with high AKT module activation scores exhibited sensitivity to a pro-apoptotic drug (fascaplysin), a result that was also not forthcoming using the full AKT signature. From all this, we can conclude that DART-CLQ was instrumental in identifying a specific AKT gene module (derived from the METABRIC discovery set), which can simultaneously predict tamoxifen resistance in ER+ breast cancer patients and sensitivity to PI3K/AKT/mTOR signaling inhibitors.

Next, we decided to validate these findings in an independent drug sensitivity screen. Since the screen from the Cancer Cell Line Encyclopedia contained few drugs [33], and these were not highly specific to the AKT/PI3K/mTOR signaling axis, we decided to use the screen generated by the Genomics Drug Sensitivity in Cancer (GDSC) project [17]. Using the same AKT gene module as before, we estimated activity scores for 39 breast cancer cell lines (Affymetrix gene expression data), which were then correlated to − log10IC50 values for 140 drugs, including rapamycin and 10 other AKT/PI3K/mTOR inhibitors. Remarkably, all 11 AKT/PI3K/mTOR inhibitors ranked above the top 65*%* correlation quantile (Wilcoxon rank sum test *P*<0.0001, Figure 6C), with four drugs (including rapamycin) ranked among the top 10*%* (i.e., above the 90*%* quantile). However, for this screen rapamycin did not rank top, presumably because for this drug there were only ten cell lines with drug sensitivity values. Thus, we sought further validation using another drug sensitivity screen [34], for which breast cancer cell-line expression data had been generated with RNA-seq, thus allowing us to also assess cross-platform reproducibility. As in the other two screens, AKT/PI3K/mTOR inhibitors were preferentially ranked at the top (Wilcoxon rank sum test *P*<10^−5^, Figure 6D), with rapamycin ranked highest.

### Biological significance of the AKT clique gene module

**Figure 7:**
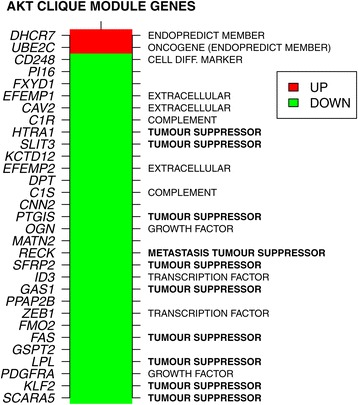
The AKT clique gene module. List of genes making up the 31-gene AKT clique module used to estimate AKT perturbation activity. Colors indicate whether they are up- or downregulated in response to AKT activation. We indicate in bold those genes that have been reported to have tumor suppressor roles in cancer. The two genes upregulated in the perturbation signature are members of the EndoPredict assay. Diff.

Given the clear clinical significance of the AKT gene module in predicting poor response to endocrine therapy *in vivo* and sensitivity to AKT/mTOR signaling inhibitors *in vitro*, we decided next to explore the biological significance of the 31 genes making up the AKT gene clique module (Additional file 1: Table S4). The AKT activation signature was originally derived by transgene expression of human *AKT1* in mouse prostate, and consisted of 187 downregulated and 172 upregulated genes [25]. Interestingly, however, only two (*DHCR7* and *UBE2C*) of the 31 genes making up the clique are upregulated in response to *AKT1* activation, the rest all being downregulated (Figure 7 and Additional file 1: Table S4). Quite remarkably, both *DHCR7* and *UBE2C* are members of the recent EndoPredict score assay, which provides prognostic information for endocrine-treated ER+/HER2 − breast cancer patients [35,36]. Among the 31 genes, a total of 6 are associated with extracellular region gene ontology terms (*HTRA1*, *EFEMP1*, *EFEMP2*, *CD248*, *SLIT3* and *LPL*). Most importantly, however, a significant proportion (34 *%*) of the downregulated genes in the module are apoptosis mediators, or have been reported to constitute putative tumor suppressors, notably *FAS*, *SFRP2*, *GAS1*, *KLF2*, *LPL*, *SCARA5*, *PTGIS*, *SLIT3* and *HTRA1* [37-49], including the metastasis suppressor *RECK* [50] (Figure 7). Thus, the identified AKT gene module links AKT signaling to inhibition of specific genes that are important in mediating apoptosis (or which have other tumor suppressive functions), consistent with the pro-survival effector function of AKT signaling.

## Discussion

In this study we have showcased a novel algorithm, called DART-CLQ, for personalized medicine, by application to the problem of endocrine resistance in ER+ breast cancer. Our aim was to identify novel subgroups of ER+ breast cancer patients who do not respond to endocrine therapy but who may benefit from alternative targeted treatments. Starting out from a large panel of 90 perturbation signatures, encompassing perturbations of many important breast cancer genes, we first identified those signatures that showed consistency and variability across the largest available breast cancer gene expression dataset [3]. We identified a total of 17 perturbation signature modules that correlated with survival in the subset of ER+ patients who had been treated either with tamoxifen or with aromatase inhibitors, in both univariate as well as multivariate analysis (Table 1). Of these 17, there were only 2 actionable signatures, both mapping to the AKT/mTOR signaling axis. One of these modules corresponded to an original perturbation signature reflecting activated AKT1. This is noteworthy for various reasons. First, AKT signaling has been proposed as one putative mechanism underlying tamoxifen and endocrine resistance in ER+ breast cancer [15,51]. Supporting this, our AKT gene module was associated with poor outcome in a meta-analysis encompassing three independent endocrine-treated ER+ cohorts (Figure 5, Table 2). Second, recent studies have not been able to demonstrate the clinical utility of PI3K mutations and other genomic aberrations within the AKT signaling pathway as markers of endocrine response in ER+ breast cancer [26-28,51]. Thus, it is significant that our AKT gene module is able to predict outcome for endocrine-treated ER+ breast cancer, when these key alterations cannot, in line with similar findings reported recently in Loi et al. [51]. Importantly, all this supports the original hypothesis that the activation status of the signaling pathway is more important than the presence of specific alterations within the pathway [8,11]. Third, AKT signaling is an actionable aberration, amenable to targeted therapy. In this regard, it is important to note that the same AKT gene module that correlates with poor outcome in endocrine-treated ER+ breast cancer, was also able to predict a high activity score in breast cancer cell lines that were particularly sensitive to treatment with PI3K/AKT/mTOR inhibitors (Figure 6). Not only does this provide another independent validation of the biological significance of our AKT gene module, but also provides a means of identifying ER+ breast cancer patients who do not respond well to endocrine therapy and who instead may benefit from additional treatment targeting the AKT/mTOR signaling axis, independently of whether key genomic or epigenomic alterations are present in this pathway or not.

There are, however, two important caveats. First, the specificity of our AKT module to predict non-response in endocrine-treated ER+ breast cancers requires further testing through a randomized clinical trial. Indeed, we note that although our AKT module did not correlate with outcome in the untreated ER+ cohorts, two of these cohorts were completely independent of the endocrine-treated ER+ cohorts, thus not allowing for a proper comparison. Second, the breast cancer cell lines used for the drug sensitivity screens are of course independent of the primary breast tumors used to show the prognostic significance of the AKT module. Thus, in future it will be important to assess the module in patient-derived cell-line models.

The biological significance of the AKT gene module identified here is supported by detailed analysis of the genes making up the module (Figure 7). Quite remarkably, the two genes upregulated in this module (*DHCR7* and *UBE2C*) are two members of the EndoPredict assay, an RT-PCR assay for predicting response to endocrine treatment in ER+/HER2 − breast cancer [35,36]. Even more interestingly, at least 34 *%* of the 29 downregulated genes (a total of 10 genes), have reported tumor suppressor functions, albeit most of these in other cancer types. Of particular interest is *GAS1*, an apoptosis inducer, which is inactivated in a wide range of different cancers [41], as well as *RECK*, a tumor and metastasis suppressor [50]. Thus, our data link AKT signaling to the downregulation and inactivation of a number of tumor suppressor genes, including mediators of apoptosis. This is highly consistent with the previously documented role of AKT signaling as an effector of pro-survival signals. It is unclear, however, how high AKT signaling activity leads to the downregulation of all of these tumor suppressors. It will be interesting to explore the underlying biological mechanisms that lead to this downregulation, as this might reveal further novel therapeutic targets.

We stress again that the novel key contribution of this work is the identification of a gene module that simultaneously correlates with a poor clinical outcome in endocrine-treated ER+ breast cancer and with sensitivity to PI3K/AKT/mTOR inhibitors. Whether the association with outcome is independent of proliferation indices, which are known to be the strongest predictors of outcome in ER+ breast cancer, is not of clinical significance. This is because proliferation indices, such as GGI [20] and MPI [52], do not present or define a specific actionable target, since they are not constructed from a single upstream perturbation that leads to the increased downstream cell proliferation. Indeed, one would not expect the outcome associations of our perturbation signatures to be independent of cell proliferation since the latter represents the phenotypic endpoint of upstream cancer driver perturbations. Supporting this, most of the prognostic associations of the perturbation signatures disappeared once we adjusted for either GGI or MPI, both of which were found to correlate with endocrine resistance in ER+ breast cancer (Additional file 1: Tables S5 and S6). To clarify, given that our goal is to (i) identify actionable perturbations driving poor outcome in endocrine-treated ER+ patients and (ii) identify the specific patients who may benefit from an alternative targeted therapy, adjustment for proliferation is not even desirable, since increased proliferation is a consequence of the upstream driver perturbation. Indeed, for our 31-gene AKT module, this was found to correlate moderately (Pearson correlation of ∼0.42) with proliferation indices (Figure 4).

Our success in identifying a potential alternative treatment for a subgroup of ER+ patients, relied not only on the overall strategy outlined here (Figure 1), but also on the specific statistical algorithm (DART-CLQ, Figure 2). We stress that naive application of perturbation signatures, which are normally derived for cell-line models, to *in vivo* data from primary cancers, may be prone to difficulties due to confounding effects related to the *in vitro* culture or the specific cell-line model considered. In our view, a step towards improving the inference and utility of these *in vitro* perturbation signatures, is first to denoise them, using a large expression set that is representative of the demographics of the cancer of interest. As shown here, not performing this denoising step (for instance, by computing Spearman rank correlations over the full perturbation signature) may lead to suboptimal inferences of perturbation activity (Figure 3 and Additional file 1: Figures S1 and S2). While our previous algorithm, DART [11], also performs a denoising step, the improved performance of DART-CLQ over DART is because DART-CLQ infers perturbation activity from a highly correlated gene module of a size that is also likely to be more biologically relevant. Indeed, the naive correlation approach would typically estimate perturbation activity using all genes in the perturbation signature, falsely assuming that all these genes are relevant in the *in vivo* context. For DART, the correlation network over which perturbation activity is estimated could still be quite large (over 100 genes). This in turn may lead to unreliable diluted activity estimates as gene correlations may fade out over larger networks. In contrast, DART-CLQ identifies approximate clique gene modules in the size range 10 to 100 genes maximum, making the resulting perturbation activity estimates much more robust and reliable (Figure 3 and Additional file 1: Figures S1 and S2).

Although DART-CLQ was successful in identifying a novel clinically relevant subtype of ER+ breast cancer, it is important to also stress the challenge of interpreting the perturbation activity score profiles. Of the 90 original perturbation signatures, many did not show significant patterns of covariability in the *in vivo* expression data, raising substantial concerns about procedures that do not attempt to denoise the signatures before estimating activity levels. We also observed a few exceptions where patterns of variation were hard to reconcile with existing knowledge. For instance, we observed a PTEN deactivation module, consisting of 20 genes, which was strongly anti-correlated with AKT activation (Pearson correlation coefficient −0.92), despite that there was only 1 gene in common between the PTEN and AKT modules. This strong anti-correlation was totally unexpected because PTEN is a suppressor of AKT signaling, so high deactivation of PTEN should correlate with high AKT activity. We verified that this apparent inconsistency was not caused by a complex clique structure in the PTEN relevance network. Much more likely, the inconsistency may reflect the poor quality of the *in vitro* PTEN perturbation signature. Supporting this view, the *PTEN* perturbation signature was not among the consistent signatures in ER − breast cancer, where *PTEN* loss is a much more frequent event [2] and where, therefore, we would have expected this signature to be prominent. This only underscores the strong need to develop a comprehensive screen of expression perturbation signatures, all generated from the same cell line and with the perturbations implemented using a protocol as uniform as possible. On a more positive note, it is comforting that the majority of the perturbation signatures showed patterns of variation and covariation that were highly consistent with previous knowledge and data. For instance, as remarked earlier, we observed strong correlations between RB inactivation and E2F3 activation signatures (Pearson >0.85), consistent with the role of RB as an inhibitor of E2F3. Similarly, the high activity of MYC signaling in luminal-B breast cancers is consistent with the frequent amplification of its locus in this subtype of breast cancer [3]. Thus, on the whole, the DART-CLQ algorithm was able to obtain activity estimates that were highly consistent, supporting previous observations [10,11].

## Conclusions

In summary, by integrating perturbation signatures with gene expression data of primary tumors and cancer cell lines with matched drug sensitivity data, we have been able to identify a novel clinically relevant subtype of ER+ breast cancer, as well as a targeted treatment (a PIK3/AKT/mTOR inhibitor) that is likely to benefit this specific patient subgroup. It will be interesting to test the predictive nature of the 31-gene AKT module in a randomized trial. The strategy implemented here, as well as the novel DART-CLQ algorithm presented in this work, will be of broad and great interest to the wider cancer community.

## Materials and methods

### Expression datasets of primary breast cancers

In this work we used the intra-sample normalized gene expression datasets, as provided by the respective publications [3,20,29-31]. For all datasets, probes mapping to the same Entrez gene ID were averaged. Inter-array normalization was performed, if deemed necessary, by using quantile normalization as implemented in the limma R package [53]. For the METABRIC study [3], the data matrix for the discovery set consisted of 24,924 genes and 774 ER+ samples, whereas the validation set consisted of the same number of genes and 651 ER+ samples. The merged METABRIC set thus consisted of 1,425 ER+ patients, of which 926 had been treated with endocrine therapy (either tamoxifen or aromatase inhibitors in combination with or without radiotherapy). Of the 1,426 ER+ patients, 360 had not received any treatment or only radiotherapy and these were classed in the untreated group.

The two other ER+ endocrine-treated cohorts were those of Loi et al. [20] (*n*=250 eligible patients) and Zhang et al. [30] (*n*=136). The two independent untreated (i.e., no endocrine treatment or chemotherapy) ER+ cohorts were those of Wang et al. [29] (*n*=209) and Miller et al. [31] (*n*=127). Further details of these cohorts can be obtained from the respective references.

### Breast cancer cell line expression datasets with drug sensitivity data

Likewise we downloaded the intra-sample normalized(if available) expression data for breast cancer cell lines that were screened for response to various drugs [16,17,34]. For Heiser’s data [16], gene expression was assessed using the Affymetrix GeneChip Human Gene 1.0 ST exon array platform. Gene-level summaries of expression were computed using AROMA and quantile normalization [16]. The intra-sample log2-normalized (AROMA) was downloaded from ArrayExpress (E-MTAB-181), and then processed further using the same method as for the primary breast cancer expression sets, resulting in a data matrix of 15,714 unique Entrez gene IDs and 56 breast cancer cell lines, of which 45 cell lines had drug sensitivity data. − log10(GI50) drug sensitivity values were available for 74 compounds.

For the GDSC data [17], gene expression was generated with the Affymetrix U133A platform. We downloaded the intra-sample normalized data available from the GDSC website [54], which was then further processed using the same procedure as before, resulting in a data matrix of 12,633 unique Entrez ID genes and 39 breast cancer cell lines. − log10(GI50) scores were available for 140 compounds.

For the Daemen et al. data [34], we used the normalized RNA-seq gene expression data available from the GEO website [55] under accession number [GEO:GSE48216]. This was then further processed using the same procedure as before, including quantile normalization and removal of low-variance genes, resulting in a data matrix of 18,295 unique Entrez ID genes and 56 breast cancer cell lines, although − log10(GI50) scores for 90 compounds were only available for 42 cell lines.

### Normal/cancer expression datasets

To validate the DART-CLQ algorithm, we used the normal/cancer gene expression dataset from Yu et al. [18]. This study profiled, using Affymetrix U133A arrays, over 300 normal/cancer samples from six tissue types, including breast (13 normals + 183 cancers), lung (12 normals + 18 cancers), thyroid (16 normals + 35 samples), liver (8 samples + 9 cancers) and colon (9 normals + 9 cancers). The intra-sample normalized data were processed in the same way as the other datasets, resulting in a dataset of 13,262 unique Entrez gene IDs and over 300 samples. We also downloaded the RNA-seq (V2) level-3 data from the TCGA [56] for the same six tissue types, i.e., using TCGA nomenclature, these were BRCA (breast cancer), LSCC & LUAD (lung cancer), THCA (thyroid cancer), LIHC (liver cancer) and COAD (colon cancer).

### Perturbation signatures from C6 class of the Molecular Signatures Database

We obtained an original list of 189 gene expression perturbation signatures from the C6 class of MSigDB (v4.0) [57]. Most of these perturbation signatures were derived from overexpression (e.g., using retroviral or transgene expression techniques) or underexpression (e.g., through RNA interference) experiments. In this work, we denote activating signatures as UP, and deactivating signatures as DN. Of the 189 perturbation signatures, 180 come in pairs, with one signature of a pair listing the genes upregulated in response to the perturbation, whilst the other lists the genes downregulated in response to the same perturbation. Thus, these 180 perturbation signatures were assembled into a list of 90 perturbation signatures, representing 90 different experiments, with each signature consisting of genes upregulated (+1) or downregulated (−1) in response to the perturbation. It follows that these perturbation signatures contain information only about the directionality of the change in response to the perturbation and not their exact fold-changes. We point out that this is not a problem, because the fold-changes for the genes making up the perturbation signatures were deemed statistically significant by the original studies in which these perturbation signatures were derived. The application of this database of 90 perturbation signatures to breast cancer is supported since many of these signatures were derived from perturbations of important breast cancer genes. The full list of 90 perturbation signatures and their definitions can be found in Additional file 1: Table S1.

### The DART-CLQ method for computing activity scores from perturbation signatures

DART-CLQ is a slight modification of our previous DART algorithm [11]. Both algorithms attempt to infer a measure of activity of a given gene expression perturbation signature (which most typically will have been derived in an *in vitro* setting), in an *in vivo* sample for which a genome-wide gene expression profile is available.

The key concept behind DART (and DART-CLQ) is a signature denoising step that aims to remove potentially confounding variation from the *in vitro* derived signature before estimating activity levels of the perturbation signature in *in vivo* samples. Briefly, the denoising step follows the DART procedure [11]: one uses a large training gene expression dataset of *in vivo* primary cancer samples to estimate pairwise correlations in gene expression for all pairs of genes in the given perturbation signature. The directionality of these correlations may be consistent or inconsistent with the predictions of the perturbation signature itself (see below for a formal definition of consistency). A consistency score can be obtained by counting the fraction of consistent pairwise correlations and statistical significance estimated using a Monte Carlo simulation procedure (1,000 Monte Carlo runs), as described by us previously [11]. Those perturbation signatures passing a *P*<0.001 threshold are deemed consistent and only these are used in further analyses. We note for these consistent perturbation signatures, those correlations that are nevertheless inconsistent with the prior information are removed from the correlation network, leaving behind a relevance correlation network of significant and consistent gene pairwise correlations. From this pruned or denoised correlation network, one then estimates a sample-specific activity level of the perturbation signature. At this point, DART and DART-CLQ differ in how the activity level is estimated. Whereas DART uses the whole pruned correlation network, DART-CLQ infers the largest cliques within the pruned correlation network and then estimates activity using only genes within a module obtained by merging the largest cliques together. Importantly, in either method, no phenotypic information is ever used in the denoising step, thus the training refers solely to the inference of a subset of the signature genes that are most relevant for obtaining the activity estimates. In more detail, the key steps of DART-CLQ are as follows:

- Assess consistency of the prior information contained in the perturbation signature with the gene pairwise correlation patterns of signature genes, as observed in the training set. Specifically, if two genes A and B are upregulated in the perturbation signature, then we would expect both of these genes to be more highly expressed in samples where the given perturbation is activated. Conversely, for a sample where the perturbation is not active or inactive, the two genes would exhibit lower expression. Thus, we would expect genes A and B to be correlated as assessed in the training gene expression dataset. Similarly, if gene A is upregulated and gene C is downregulated in the perturbation signature, then we would expect A and C to be anti-correlated, assuming of course that this perturbation signature exhibits relevant variation across the samples of the *in vivo* gene expression dataset. Importantly, using Monte Carlo permutations, we can evaluate the statistical significance of an overall consistency score obtained as the number of gene pairs for which the observed correlation is consistent with that predicted by the perturbation signature [11].
- For those signatures that exhibit statistically significant consistency levels, we prune their correlation relevance networks to remove edges/correlations that are inconsistent with the prior information. The rest of the signatures, which are inconsistent, are ignored, since the lack of statistical consistency means that the prior information contained in the signature is not seen to explain variation in the *in vivo* training data, so there is no statistical justification for computing activity scores for these signatures.
- Given the pruned correlation network of a consistent perturbation signature, where each edge (representing a gene pair) now represents a statistically significant pairwise correlation for which the directionality agrees with that of the prior information, we now identify the largest clique(s) in this network. In practice, these cliques often exhibit very large overlaps with each other. Thus, we construct the union of all genes making up the largest cliques, and extract the relevance network of this subset of genes. Although the resulting network may not be a clique, it will be a highly connected subnetwork approximating a clique, which we call a clique module.
- Given this gene clique module, the activity score is now computed exactly as in the previous DART algorithm [11]. Specifically, we construct a weighted average of the z-score normalized expression profiles of the genes making up the module, with the weights, *σ*_*g*_, being +1 for genes that were upregulated in the original perturbation signature, while being −1 for genes that were downregulated. This scheme thus allows us to keep track of the directionality of the activity levels. Thus, in a sample were an upregulated gene is highly expressed, its z-score will be positive, and the prior weight being also positive means that this gene makes a positive contribution to the activity score, as required. Likewise a gene predicted to be downregulated by the perturbation and that is lowly expressed in that same sample, will have a negative z-score as well as a negative weight, thus also contributing positively to the score, as required. In terms of equations:
  (1) $$ S_{s}^{(p)} \propto \sum_{g\in \text{CLQ-MOD}(p)}{k_{g}\sigma^{(p)}_{g}z_{gs}}  $$
  where $S_{s}^{(p)}$ denotes the activity score of perturbation signature *p* in sample *s*, $\sigma ^{(p)}_{g}=1$ (−1) if gene *g* is upregulated (downregulated) in perturbation signature *p*, *z*_*gs*_ denotes the z-score normalized gene expression level of gene *g* in sample *s* and where CLQ-MOD(*p*) denotes the clique module inferred for perturbation signature *p*. In the above, *k*_*g*_ denotes the connectivity or degree of gene *g* in the clique module, although this will vary insignificantly within the module, as most genes will have very similar if not identical degree.

### Construction and testing of random signatures

To provide independent tests of the statistical significance of the prognostic associations reported in Table 1, we used two separate strategies based on the notion of random signatures. In one approach, we constructed 90 random perturbation signatures, matched to the same size and distribution of up- and downregulated genes of the 90 MSigDB C6 perturbation signatures. These 90 random signatures were processed in the same way, by running them through DART-CLQ, to assess whether their observed correlative patterns are consistent with those predicted by the signature. In this analysis, none of the 90 random signatures achieved a level of significance of their consistency score (Monte Carlo test *P*<0.001), compared to 57 of the 90 MSigDB C6 signatures that did pass this level of significance. Thus, no further Cox regressions of the 90 random signatures were required since all random signatures already failed the consistency score test built within DART-CLQ. Results were unchanged under repeated constructions of 90 random signatures.

In the second approach, we followed the strategy of Venet et al. [22] to test expression signatures that, in principle, should be unrelated to breast cancer prognosis. To construct such expression signatures we used a large expression dataset of 353 normal tissue specimens from 65 different anatomical sites [24], to derive signatures of differential expression between tissue types (anatomical sites) that are unrelated to breast epithelial cells. This set of sites included skeletal muscle, spinal cord, prostate gland, lymph nodes, liver, coronary artery, testes, tonsil and four different sites from the brain (hypothalamus, midbrain, hippocampus and cerebral cortex). For each of these sites, we had at least three independent samples. From these 12 anatomical sites, we performed 66 (=12×11×0.5) pairwise differential expression analysis comparisons, selecting in each case the top 337 differentially expressed genes, as inferred using limma [53]. We selected the top 337, because this was the average size of the MSigDB C6 perturbation signatures. As in Venet et al., we reasoned that these 66 anatomical-site-specific signatures should not exhibit as strong prognostic association as our 17 DART-CLQ modules, if the latter represent genuine associations. Thus, we ran the 66 signatures through DART-CLQ, which identified a subset of 63 with significant consistency scores. For these 63, Cox regressions were performed in the same endocrine-treated ER+ METABRIC subset and that resulted in 12 attaining Cox *P* values stronger than the largest *P* value of the 17 DART-CLQ modules. We thus estimated an empirical FDR of approximately 12/63≃0.19, in close agreement with the Benjamini–Hochberg estimate of 0.15.

### Availability

The DART algorithm is freely available from Bioconductor [58]. DART-CLQ represents a minor modification of DART, and an R script implementing the modified DART-CLQ method for estimating perturbation activity estimates is freely available as Additional file 2 and from SourceForge [59] under the MIT license.

## Additional files

Additional file 1
**Contains all supplementary figures, supplementary tables and their respective legends.**

Additional file 2
**R script to estimate perturbation activity using DART-CLQ.**

## Abbreviations

DART: Denoising algorithm with relevance network topology
ER+: Estrogen receptor positive
ER −: Estrogen receptor negative
FDR: False discovery rate
GDSC: Genomics Drug Sensitivity in Cancer Project
GGI: Genome grade index
MPI: Molecular prognostic index
MSigDB: Molecular Signatures Database
RNA-seq: RNA sequencing
TCGA: The Cancer Genome Atlas
