# Supplementary material for: Denoising perturbation signatures reveal an actionable AKT-signaling gene module underlying a poor clinical outcome in endocrine-treated ER+ breast cancer
Source: Genome Biol. 2015 Apr 2;16(1):61. doi: 10.1186/s13059-015-0630-4 (PMC4399757; doi:10.1186/s13059-015-0630-4)
Supplement: Additional file 1 — Contains all supplementary figures, supplementary tables and their respective legends. [file 13059_2015_630_MOESM1_ESM.pdf]

Additional file 1

Denoising perturbation signatures reveals an actionable AKT-signaling gene module underlying a poor clinical outcome in endocrine treated ER+ breast cancer

Andrew E. Teschendorff [*,1,2,3] , Linlin Li [1,3] and Zhen Yang[1]

[1] CAS-MPG Partner Institute for Computational Biology, Chinese Academy of Sciences, Shanghai Institute for Biological Sciences,320 Yue Yang Road, 200031 Shanghai, China. [2]Statistical Genomics Group, Paul O'Gorman Building, UCL Cancer Institute, University College London, 72 Huntley Street, London WC1E 6BT, United Kingdom.
[3] Equal Contribution.

*Corresponding Author: andrew.teschendorff@ucl.ac.uk, or andrew@picb.ac.cn

SUPPLEMENTARY FIGURES:

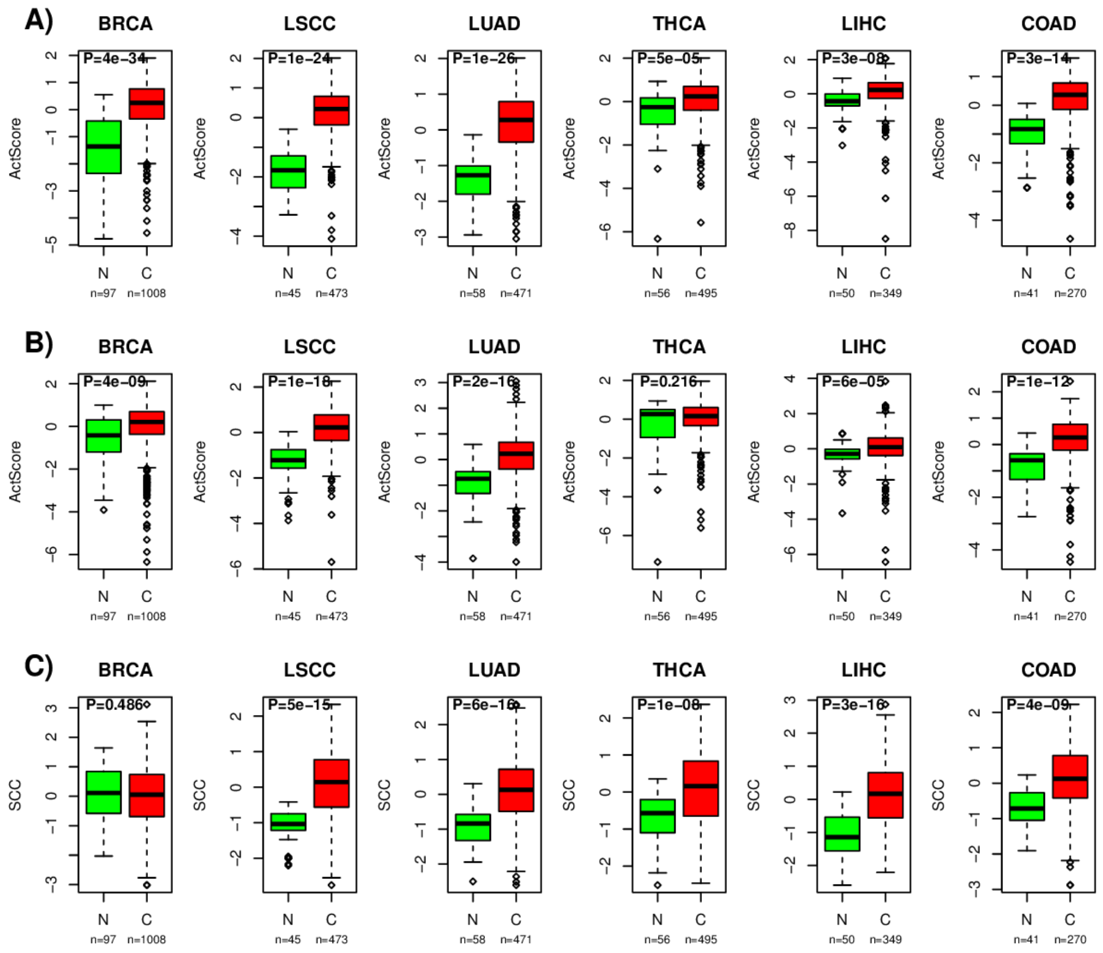

Figure S1: Validation of the p53 signature activity score in TCGA data sets encompassing normal and cancer tissue. Boxplots compare the activity of the p53 deactivation signature, as evaluated using **A)** DART-CLQ, **B)** DART, **C)** Spearman rank correlation coefficients (SCC), across normal (N) and cancer (C) tissues using mRNA RNA-SeqV2 expression data from the TCGA. Number of samples in each group are indicated below boxplots. P-values are from a Wilcoxon rank sum test. Observe how DART and SCC fail to yield a higher score in the thyroid and breast cancer sets, respectively.

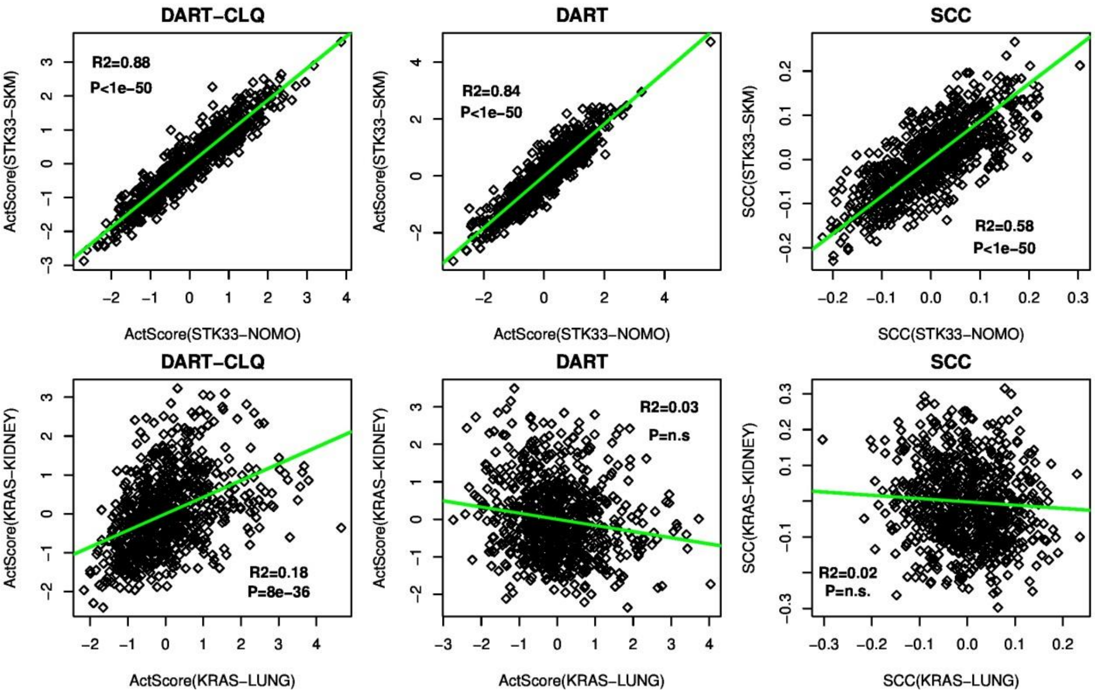

Figure S2: Validation and comparison of the DART-CLQ method to DART and Spearman rank correlation coefficients (SCC) to infer perturbation signature activity scores. Top panels show the predicted activity scores for two independent STK33 perturbation signatures (in the ER+ breast cancer Metabric set) as assessed using DART-CLQ, DART and Spearman correlations. Lower panels compare the predicted activity scores for two KRAS perturbation signatures derived in different tissue types (kidney and lung), also in the ER+ Metabric set. R^2 and P-values of a linear regression are given. For negative regression coefficients, the significance of a positive correlation is indicated accordingly as “n.s” (not significant).

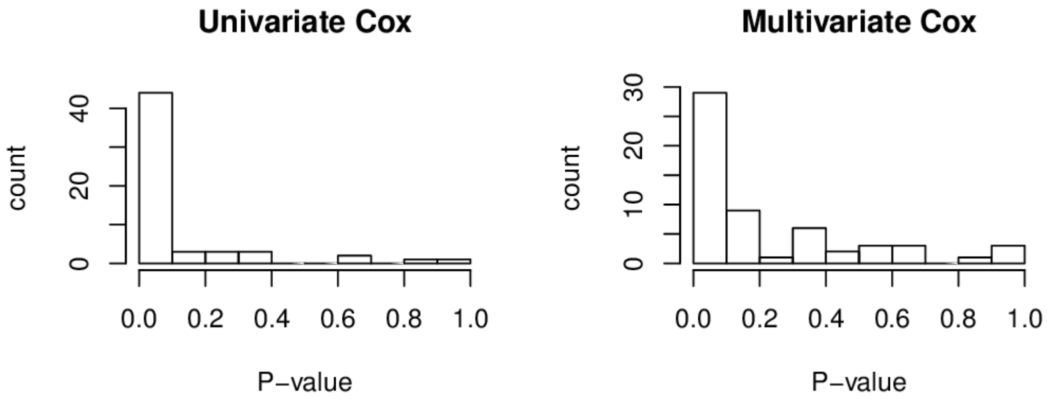

Figure S3: Histograms of Cox-regression P-values (for overall survival) for the 57 DART-CLQ modules in the endocrine treated ER+ subset of the METABRIC cohort. Left panel is for the P-values from the univariate analysis, right panel for the P-values from the multivariate Cox-regression including stage, grade and tumour size. Note how P-values are skewed

towards small values.

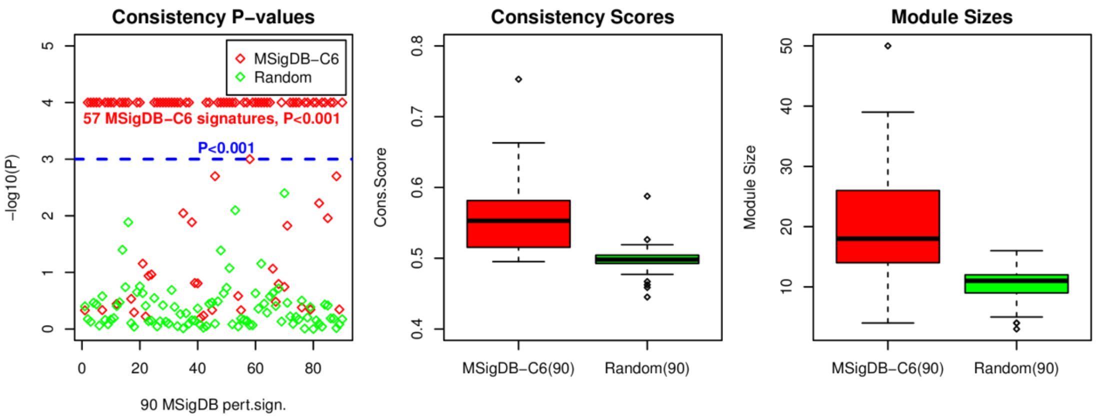

Figure S4: Left panel: depicts the –log10(P-values) of the DART-CLQ consistency score for the 90 MSigDB C6 perturbation signatures (red). The P-value of the consistency score describes how consistent the observed (in-vivo) correlative patterns of the perturbation signature genes are with those predicted by the in-vitro perturbation signature itself. A total of 57 signatures pass the threshold of 0.001 (indicated in blue). In contrast, none of 90 randomly constructed signatures, matched for size and up/down regulation distribution, passed this same threshold. **Middle panel:** boxplot of the DART-CLQ consistency scores for the 90 MSigDB C6 signatures compared to those of the random set. Observe how these scores hover around 0.5 for the random set of signatures, as required. **Right panel:** boxplot of the DART-CLQ module sizes of the 90 MSigDB C6 signatures compared to those of the random set. Observe how the MSigDB perturbation signatures lead to larger modules than expected by random chance.

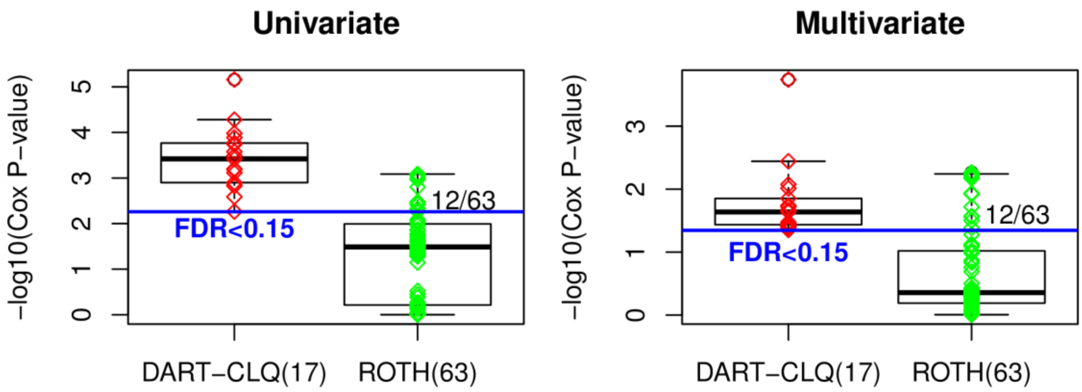

Figure S5: Boxplots of –log10(P-values) from univariate and multivariate (adjusted for grade, stage and tumour size) Cox-regressions for the 17 DART-CLQ modules (their activity scores were correlated to survival) that pass an FDR<0.15 in both univariate and multivariate analysis. For comparison, we also show the corresponding Cox-regression P-values obtained for 63 unrelated signatures derived from the Roth et al data set (see paper for reference) by comparing normal tissues from different anatomical sites (Materials and Methods). The

number of these 63 signatures that pass the P-value threshold corresponding to our FDR<0.15 estimate are indicated and was 12 in both univariate and multivariate analysis.

SUPPLEMENTARY TABLES:

Pert.Sign	Class	n(UP)	n(DN)	ABERRATION_BC
GLI1_UP.V1	ACTIVATING	27	29	AMPLIFIED
E2F1_UP.V1	ACTIVATING	189	193	AMPLIFIED
EGFR_UP.V1	ACTIVATING	193	196	AMPLIFIED
ERB2_UP.V1	ACTIVATING	192	197	AMPLIFIED, MUTATED
GCNP_SHH_UP_EARLY.V1	ACTIVATING	174	169	AMPLIFIED
GCNP_SHH_UP_LATE.V1	ACTIVATING	183	180	AMPLIFIED
RAPA_EARLY_UP.V1	ACTIVATING	181	189	NA
CYCLIN_D1_KE_.V1	ACTIVATING	190	194	AMPLIFIED
CYCLIN_D1_UP.V1	ACTIVATING	188	191	AMPLIFIED
CSR_EARLY_UP.V1	ACTIVATING	164	154	NA
CSR_LATE_UP.V1	ACTIVATING	172	170	NA
AKT_UP_MTOR_DN.V1	DUAL	184	183	PIK3CA MUT.,AKT1 GAINS
AKT_UP.V1	ACTIVATING	172	187	PIK3CA MUT.,AKT1 GAINS
MTOR_UP.V1	ACTIVATING	170	184	NA
PIGF_UP.V1	ACTIVATING	191	194	NA
VEGF_A_UP.V1	ACTIVATING	196	193	NA
BCAT_GDS748	ACTIVATING	48	47	NA
BCAT.100_UP.V1	ACTIVATING	49	45	NA
ATF2_S_UP.V1	ACTIVATING	193	187	MUTATED
ATF2_UP.V1	ACTIVATING	192	187	MUTATED
WNT_UP.V1	ACTIVATING	180	170	NA
ATM_DN.V1	DEACTIVATING	146	149	LOSS/DELET.
P53_DN.V2	DEACTIVATING	148	145	MUTATED
RELA_DN.V1	DEACTIVATING	149	141	NA
P53_DN.V1	DEACTIVATING	194	192	MUTATED
BCAT_BILD_ET_AL	ACTIVATING	49	46	NA
E2F3_UP.V1	ACTIVATING	196	183	AMPLIFIED
MYC_UP.V1	ACTIVATING	186	182	AMPLIFIED
SRC_UP.V1	ACTIVATING	188	179	AMPLIFIED
SNF5_DN.V1	DEACTIVATING	177	164	NA
CAMP_UP.V1	ACTIVATING	200	200	NA
LTE2_UP.V1	ACTIVATING	190	196	NA
MEK_UP.V1	ACTIVATING	196	196	LOSS/DELETION
RAF_UP.V1	ACTIVATING	196	194	NA
PRC1_BMI_UP.V1	ACTIVATING	189	191	AMPLIFIED/MUTATEED
PRC2_EDD_UP.V1	ACTIVATING	193	194	AMPLIFIED/DELETION
PRC2_EZH2_UP.V1	ACTIVATING	195	194	mRNA CHANGE
PRC2_SUZ12_UP.V1	ACTIVATING	194	191	AMPLIFIED/DELETION

JNK_DN.V1	DEACTIVATING	192	191	NA
BRCA1_DN.V1	DEACTIVATING	141	143	INACT. MUTATED
CTIP_DN.V1	DEACTIVATING	138	141	MUTATION/DELETION
PKCA_DN.V1	DEACTIVATING	170	167	NA
MTOR_UP.N4.V1	ACTIVATING	196	193	NA
PTEN_DN.V2	DEACTIVATING	143	144	LOSS
DCA_UP.V1	ACTIVATING	191	193	NA
ESC_J1_UP_EARLY.V1	ACTIVATING	182	178	NA
ESC_J1_UP_LATE.V1	ACTIVATING	191	186	NA
ESC_V6.5_UP_EARLY.V1	ACTIVATING	170	172	NA
ESC_V6.5_UP_LATE.V1	ACTIVATING	190	186	NA
ALK_DN.V1	DEACTIVATING	145	148	NA
BMI1_DN_MEL18_DN.V1	DEACTIVATING	145	147	AMPLIFIED, MUTATED
BMI1_DN.V1	DEACTIVATING	147	144	AMPLIFIED
MEL18_DN.V1	DEACTIVATING	141	148	MUTATED, DELETION
PTEN_DN.V1	DEACTIVATING	191	187	LOSS
NOTCH_DN.V1	DEACTIVATING	193	189	AMPLIFIED
EIF4E	ACTIVATING	100	100	DELETION
CRX_DN.V1	DEACTIVATING	136	134	NA
CRX_NRL_DN.V1	DEACTIVATING	140	130	NA
NRL_DN.V1	DEACTIVATING	136	134	NA
RB_DN.V1	DEACTIVATING	137	126	LOSS/DELET.
RB_P107_DN.V1	DEACTIVATING	140	128	LOSS/DELET.
RB_P130_DN.V1	DEACTIVATING	133	139	LOSS/DELET.
RPS14_DN.V1	DEACTIVATING	192	187	NA
IL15_UP.V1	ACTIVATING	192	190	NA
IL2_UP.V1	ACTIVATING	192	196	NA
IL21_UP.V1	ACTIVATING	193	187	NA
PDGF_ERK_DN.V1	DEACTIVATING	147	149	NA
PDGF_UP.V1	ACTIVATING	146	142	NA
TGFB_UP.V1	ACTIVATING	192	192	GAINS
YAP1	ACTIVATING	47	48	AMPLIFIED, DELETION
SIRNA_EIF4GI	DEACTIVATING	95	99	NA
HOXA9_DN.V1	DEACTIVATING	194	195	PROMOTER METHYL.
STK33	DEACTIVATING	293	289	DIFF. METHYLATION
STK33_NOMO	DEACTIVATING	294	292	DIFF. METHYLATION
STK33_SKM	DEACTIVATING	290	288	DIFF. METHYLATION
KRAS.AMP.LUNG_UP.V1	ACTIVATING	144	146	AMPLIFIED, MUTATED
KRAS.DF.V1	ACTIVATING	193	194	AMPLIFIED, MUTATED
TBK1.DF	DEACTIVATING	290	287	NA
TBK1.DN.48HRS	DEACTIVATING	50	50	NA
JAK2_DN.V1	DEACTIVATING	188	173	NA
KRAS.300_UP.V1	ACTIVATING	146	143	AMPLIFIED
KRAS.50_UP.V1	ACTIVATING	48	49	AMPLIFIED

KRAS.600_UP.V1	ACTIVATING	287	289	AMPLIFIED
KRAS.600.LUNG.BREAST_UP.V1	ACTIVATING	288	289	AMPLIFIED
KRAS.BREAST_UP.V1	ACTIVATING	146	145	AMPLIFIED
KRAS.KIDNEY_UP.V1	DEACTIVATING	145	142	AMPLIFIED
KRAS.LUNG_UP.V1	ACTIVATING	141	145	AMPLIFIED
KRAS.LUNG.BREAST_UP.V1	ACTIVATING	145	145	AMPLIFIED
KRAS.PROSTATE_UP.V1	ACTIVATING	143	144	AMPLIFIED
LEF1_UP.V1	ACTIVATING	195	190	AMPLIFIED

Table S1: List of the 90 perturbation signatures from the C6 signature class from MSigDB, indicating whether activating or deactivating, number of up and downregulated genes making up the perturbation signature, and whether the perturbed gene is altered in breast cancer, and if so, how.

Pert.Sign.	t(LumB-LumA)	P
GCNP_SHH_UP_LATE.V1	27.36	4.00E-106
VEGF_A_UP.V1	-27.43	2.00E-103
GCNP_SHH_UP_EARLY.V1	26.23	2.00E-98
PRC2_EZH2_UP.V1	25.62	4.00E-97
E2F1_UP.V1	25.89	3.00E-95
RB_P130_DN.V1	23.92	1.00E-87
MTOR_UP.V1	22.84	7.00E-85
PRC2_EDD_UP.V1	23.21	3.00E-84
SRC_UP.V1	-22.1	2.00E-79
RB_P107_DN.V1	22.78	2.00E-78
RB_DN.V1	22.81	3.00E-77
CSR_LATE_UP.V1	20.54	2.00E-72
ATF2_UP.V1	19.19	1.00E-64
E2F3_UP.V1	19.72	5.00E-64
ATF2_S_UP.V1	18.14	1.00E-59
MYC_UP.V1	13.54	1.00E-35
KRAS.600_UP.V1	-13.07	6.00E-35
ESC_J1_UP_LATE.V1	-12.11	4.00E-30
AKT_UP.V1	11.98	6.00E-30
KRAS.600.LUNG.BREAST_UP.V1	-11.69	5.00E-29
KRAS.KIDNEY_UP.V1	-11.56	3.00E-28
PTEN_DN.V2	-10.97	7.00E-26
KRAS.300_UP.V1	-10.82	3.00E-25
TGFB_UP.V1	-10.26	8.00E-23
ESC_V6.5_UP_LATE.V1	-9.96	1.00E-21
ESC_V6.5_UP_EARLY.V1	9.32	4.00E-19
EGFR_UP.V1	-9.22	4.00E-19

CAMP_UP.V1	8.75	3.00E-17
MTOR_UP.N4.V1	8.27	9.00E-16
LEF1_UP.V1	-8.08	4.00E-15
NRL_DN.V1	7.75	4.00E-14
CSR_EARLY_UP.V1	7.56	2.00E-13
RAF_UP.V1	-7.22	2.00E-12
STK33	-6.82	2.00E-11
TBK1.DF	-6.8	3.00E-11
KRAS.DF.V1	-6.68	6.00E-11
CRX_DN.V1	6.18	1.00E-09
P53_DN.V1	5.8	1.00E-08
ERB2_UP.V1	-5.74	2.00E-08
JAK2_DN.V1	4.96	9.30E-07
MEK_UP.V1	-4.56	6.38E-06
LTE2_UP.V1	-4.51	8.10E-06
IL15_UP.V1	-3.88	0.00011662
STK33_SKM	-3.78	0.00017238
STK33_NOMO	-3.61	0.00033671
MEL18_DN.V1	-3.42	0.00066501
BCAT_BILD_ET_AL	3.32	0.00095671
BMI1_DN_MEL18_DN.V1	-2.77	0.00579313
HOXA9_DN.V1	-1.49	0.1361676
SNF5_DN.V1	1.34	0.18130114
IL2_UP.V1	1.32	0.18658941
PIGF_UP.V1	1.22	0.22147002
CYCLIN_D1_UP.V1	-1.2	0.23162966
ALK_DN.V1	-1.17	0.2423551
RPS14_DN.V1	0.91	0.36114869
KRAS.LUNG_UP.V1	0.76	0.44628672
CYCLIN_D1_KE_.V1	0.4	0.69153008

Table S2: The 57 highly variable and consistent perturbation signatures, ranked according to strength of association with luminal A/B subtype status within the ER+ subset of the Metabric discovery set. T-test statistics and P-values are given. Positive t-statistics indicate larger activity scores in lum-B tumours.

PertSign	HR(95%CI)	P	n
E2F1_UP.V1	1.2 (1.09-1.32)	0.00026865	926
EGFR_UP.V1	0.93 (0.83-1.03)	0.14280116	926
ERB2_UP.V1	1.1 (0.99-1.22)	0.06926851	926
GCNP_SHH_UP_EARLY.V1	1.19 (1.08-1.31)	0.00034212	926
GCNP_SHH_UP_LATE.V1	1.2 (1.09-1.32)	0.00022931	926
CYCLIN_D1_KE_.V1	1.14 (1.04-1.25)	0.00543566	926
CYCLIN_D1_UP.V1	1.11 (1.01-1.22)	0.02938477	926
CSR_EARLY_UP.V1	0.91 (0.82-1)	0.04174004	926
CSR_LATE_UP.V1	1.19 (1.08-1.32)	0.0006549	926
AKT_UP.V1	1.18 (1.07-1.31)	0.00144295	926
MTOR_UP.V1	1.2 (1.09-1.33)	0.00035448	926
PIGF_UP.V1	0.86 (0.78-0.94)	0.00126015	926
VEGF_A_UP.V1	0.85 (0.77-0.94)	0.00096113	926
ATF2_S_UP.V1	1.2 (1.08-1.32)	0.00063269	926
ATF2_UP.V1	1.21 (1.09-1.34)	0.00026456	926
P53_DN.V1	1.13 (1.02-1.24)	0.01777418	926
BCAT_BILD_ET_AL	1.05 (0.95-1.15)	0.37116891	926
E2F3_UP.V1	1.21 (1.1-1.33)	5.25E-05	926
MYC_UP.V1	1.14 (1.04-1.26)	0.00682646	926
SRC_UP.V1	0.83 (0.75-0.91)	0.00018118	926
SNF5_DN.V1	1.01 (0.92-1.11)	0.84975947	926
CAMP_UP.V1	1.13 (1.03-1.25)	0.01096439	926
LTE2_UP.V1	0.91 (0.82-1)	0.04007185	926
MEK_UP.V1	0.91 (0.83-1)	0.04947735	926
RAF_UP.V1	0.9 (0.82-0.99)	0.03336568	926
PRC2_EDD_UP.V1	1.21 (1.1-1.33)	0.00012952	926
PRC2_EZH2_UP.V1	1.18 (1.07-1.3)	0.00086371	926
MTOR_UP.N4.V1	1.19 (1.08-1.32)	0.00076847	926
PTEN_DN.V2	0.84 (0.76-0.94)	0.00148631	926
ESC_J1_UP_LATE.V1	0.88 (0.8-0.97)	0.01158877	926
ESC_V6.5_UP_EARLY.V1	1.06 (0.96-1.16)	0.25857532	926
ESC_V6.5_UP_LATE.V1	0.85 (0.77-0.94)	0.00154668	926
ALK_DN.V1	0.95 (0.86-1.05)	0.31617563	926
BMI1_DN_MEL18_DN.V1	0.95 (0.86-1.05)	0.30365009	926
MEL18_DN.V1	0.98 (0.88-1.07)	0.61149325	926
CRX_DN.V1	1.13 (1.02-1.24)	0.01426345	926
NRL_DN.V1	1.16 (1.05-1.28)	0.00481543	926
RB_DN.V1	1.09 (0.99-1.19)	0.06993838	926
RB_P107_DN.V1	1.2 (1.09-1.31)	0.00017122	926
RB_P130_DN.V1	1.21 (1.1-1.34)	0.00010457	926
RPS14_DN.V1	0.98 (0.89-1.08)	0.63901056	926
IL15_UP.V1	0.93 (0.84-1.03)	0.16634198	926

IL2_UP.V1	1 (0.9-1.1)	0.94479211	926
TGFB_UP.V1	0.9 (0.81-0.99)	0.03389235	926
HOXA9_DN.V1	0.94 (0.85-1.04)	0.24400347	926
STK33	0.88 (0.79-0.97)	0.0107324	926
STK33_NOMO	0.9 (0.82-1)	0.0409039	926
STK33_SKM	0.93 (0.84-1.03)	0.1466548	926
KRAS.DF.V1	0.91 (0.82-1)	0.04426218	926
TBK1.DF	0.88 (0.8-0.97)	0.01233058	926
JAK2_DN.V1	0.89 (0.82-0.97)	0.00745289	926
KRAS.300_UP.V1	0.78 (0.7-0.87)	6.93E-06	926
KRAS.600_UP.V1	0.82 (0.74-0.92)	0.0003805	926
KRAS.600.LUNG.BREAST_UP.V1	0.84 (0.75-0.94)	0.00258598	926
KRAS.KIDNEY_UP.V1	0.83 (0.75-0.92)	0.0003261	926
KRAS.LUNG_UP.V1	0.95 (0.86-1.05)	0.28334428	926
LEF1_UP.V1	0.9 (0.82-0.99)	0.03758052	926

Table S3: Univariate Cox regression analysis of the 57 consistent perturbation signatures in the endocrine treated ER+ patients of the merged Metabric set. We give the Hazard Ratio (HR), 95% CI, log-rank test P-value and number of samples.

Symbol	EntrezID	UP(1)/DN(-1)	Description
DHCR7	1717	1	7-dehydrocholesterol reductase
UBE2C	11065	1	ubiquitin-conjugating enzyme E2C
CD248	57124	-1	CD248 molecule, endosialin
PI16	221476	-1	peptidase inhibitor 16
FXYD1	5348	-1	FXYD domain containing ion transport regulator 1
EFEMP1	2202	-1	EGF containing fibulin-like extracellular matrix protein 1
CAV2	858	-1	caveolin 2
C1R	715	-1	complement component 1, r subcomponent
**HTRA1**	5654	-1	HtrA serine peptidase 1
**SLIT3**	6586	-1	slit homolog 3 (Drosophila)
KCTD12	115207	-1	potassium channel tetramerisation domain containing 12
EFEMP2	30008	-1	EGF containing fibulin-like extracellular matrix protein 2
DPT	1805	-1	dermatopontin
C1S	716	-1	complement component 1, s subcomponent
CNN2	1265	-1	calponin 2
**PTGIS**	5740	-1	prostaglandin I2 (prostacyclin) synthase
OGN	4969	-1	osteoglycin
MATN2	4147	-1	matrilin 2
**RECK**	8434	-1	reversion-inducing-cysteine-rich protein with kazal motifs
**SFRP2**	6423	-1	secreted frizzled-related protein 2

ID3	3399	-1	inhibitor of DNA binding 3, dominant negative helix-loop-helix protein
**GAS1**	2619	-1	growth arrest-specific 1
PPAP2B	8613	-1	phosphatidic acid phosphatase type 2B
ZEB1	6935	-1	zinc finger E-box binding homeobox 1
FMO2	2327	-1	flavin containing monooxygenase 2 (non-functional)
**FAS**	355	-1	Fas (TNF receptor superfamily, member 6)
GSPT2	23708	-1	G1 to S phase transition 2
**LPL**	4023	-1	lipoprotein lipase
PDGFRA	5156	-1	platelet-derived growth factor receptor, alpha polypeptide
**KLF2**	10365	-1	Kruppel-like factor 2 (lung)
**SCARA5**	286133	-1	scavenger receptor class A, member 5 (putative)

Table S4: The 31 genes making up the AKT gene clique module, used to infer activity of the corresponding AKT perturbation signature. We provide the Gene Symbol, Entrez gene ID, whether it is up or downregulated in response to AKT1 activation, and gene description. The genes in boldface have reported tumour suppressor functions. The two upregulated genes are Endopredict members (see main text).

	HR	P	n
MPI(UV)	2.06 (1.42-2.99)	0.0001	926
MPI(MV)	1.6 (1.01-2.55)	0.048	685
GGI(UV)	1.61 (1.26-2.06)	0.0002	926
GGI(MV)	1.35 (0.98-1.85)	0.065	685

Table S5: Cox-regression results of the Molecular Prognostic Index (MPI) of Teschendorff et al (Genome Biol. 2006) and the Genomic Grade Index (GGI) of Loi et al (JCO 2007) in the combined METABRIC set. Univariate analysis (UV) and multivariate analysis (MV) adjusted for size, stage and grade. We give the hazard ratio (HR) + 95%CI, the log-rank test P-value, and the number of samples.

	**HR(95%CI) adj.MPI**	**P adj.MPI**	**HR(95%CI) adj.GGI**	**P adj.GGI**	**n**
E2F1_UP.V1	1.06 (0.87-1.29)	0.572	1.07 (0.87-1.31)	0.5481	926
EGFR_UP.V1	1.05 (0.93-1.19)	0.4633	0.98 (0.88-1.09)	0.7154	926
ERB2_UP.V1	1.17 (1.06-1.3)	0.0028	1.15 (1.04-1.28)	0.0082	926
GCNP_SHH_UP_EARLY.V1	1.05 (0.87-1.27)	0.6271	1.06 (0.87-1.29)	0.5863	926
GCNP_SHH_UP_LATE.V1	1.06 (0.84-1.33)	0.6321	1.08 (0.89-1.32)	0.415	926
CYCLIN_D1_KE_.V1	1.1 (1-1.21)	0.048	1.13 (1.04-1.24)	0.0069	926
CYCLIN_D1_UP.V1	1.08 (0.99-1.19)	0.0927	1.12 (1.02-1.23)	0.0167	926
CSR_EARLY_UP.V1	0.85 (0.77-0.94)	0.001	0.84 (0.77-0.93)	0.0008	926
CSR_LATE_UP.V1	1.05 (0.87-1.25)	0.6223	1.09 (0.96-1.25)	0.1958	926
AKT_UP.V1	1.07 (0.93-1.22)	0.3372	1.11 (0.99-1.24)	0.0683	926
MTOR_UP.V1	1.07 (0.89-1.29)	0.4742	1.1 (0.95-1.27)	0.2071	926
PIGF_UP.V1	0.87 (0.8-0.96)	0.0041	0.86 (0.79-0.94)	0.0014	926

VEGF_A_UP.V1	1.02 (0.84-1.24)	0.8503	1.03 (0.83-1.27)	0.8133	926
ATF2_S_UP.V1	1.06 (0.89-1.25)	0.5334	1.1 (0.96-1.25)	0.162	926
ATF2_UP.V1	1.09 (0.91-1.3)	0.3623	1.12 (0.98-1.28)	0.1076	926
P53_DN.V1	1.04 (0.93-1.16)	0.5019	1.09 (0.99-1.21)	0.0815	926
BCAT_BILD_ET_AL	0.97 (0.87-1.08)	0.5463	1.02 (0.93-1.13)	0.6288	926
E2F3_UP.V1	1.13 (0.95-1.35)	0.161	1.14 (0.98-1.32)	0.0961	926
MYC_UP.V1	1.01 (0.89-1.15)	0.8424	1.03 (0.92-1.17)	0.5845	926
SRC_UP.V1	0.92 (0.76-1.11)	0.3661	0.9 (0.78-1.05)	0.1688	926
SNF5_DN.V1	1.04 (0.95-1.15)	0.399	0.99 (0.9-1.09)	0.7958	926
CAMP_UP.V1	1.03 (0.91-1.16)	0.6395	1.08 (0.98-1.2)	0.1352	926
LTE2_UP.V1	0.94 (0.86-1.04)	0.2286	0.95 (0.86-1.04)	0.2744	926
MEK_UP.V1	0.95 (0.86-1.04)	0.2812	0.95 (0.86-1.05)	0.3192	926
RAF_UP.V1	0.99 (0.88-1.1)	0.7979	0.93 (0.84-1.03)	0.18	926
PRC2_EDD_UP.V1	1.1 (0.92-1.33)	0.2955	1.12 (0.96-1.31)	0.1659	926
PRC2_EZH2_UP.V1	0.99 (0.81-1.21)	0.9223	1.03 (0.86-1.23)	0.7576	926
MTOR_UP.N4.V1	1.11 (0.98-1.25)	0.0984	1.14 (1.02-1.27)	0.0177	926
PTEN_DN.V2	0.93 (0.81-1.06)	0.276	0.89 (0.8-1)	0.0504	926
ESC_J1_UP_LATE.V1	0.99 (0.88-1.12)	0.9167	0.95 (0.85-1.06)	0.3283	926
ESC_V6.5_UP_EARLY.V1	0.93 (0.83-1.05)	0.2396	0.99 (0.89-1.1)	0.8614	926
ESC_V6.5_UP_LATE.V1	0.93 (0.83-1.04)	0.2071	0.9 (0.81-1)	0.049	926
ALK_DN.V1	0.99 (0.9-1.1)	0.8974	0.94 (0.85-1.04)	0.2023	926
BMI1_DN_MEL18_DN.V1	1.01 (0.91-1.12)	0.8376	0.96 (0.87-1.06)	0.3948	926
MEL18_DN.V1	1.04 (0.94-1.15)	0.4447	0.99 (0.9-1.09)	0.8352	926
CRX_DN.V1	1.04 (0.93-1.16)	0.4834	1.09 (0.99-1.2)	0.0903	926
NRL_DN.V1	1.05 (0.93-1.19)	0.3994	1.1 (0.99-1.23)	0.0662	926
RB_DN.V1	0.87 (0.75-1.01)	0.0627	0.85 (0.73-0.99)	0.0364	926
RB_P107_DN.V1	1.09 (0.92-1.28)	0.3286	1.09 (0.92-1.3)	0.3238	926
RB_P130_DN.V1	1.11 (0.9-1.37)	0.3112	1.12 (0.94-1.35)	0.1983	926
RPS14_DN.V1	1.01 (0.92-1.12)	0.7744	0.96 (0.87-1.06)	0.4365	926
IL15_UP.V1	1.01 (0.91-1.13)	0.8471	0.95 (0.86-1.05)	0.3069	926
IL2_UP.V1	1.02 (0.92-1.12)	0.7179	0.97 (0.88-1.07)	0.4892	926
TGFB_UP.V1	1.02 (0.9-1.16)	0.7666	0.96 (0.86-1.07)	0.4512	926
HOXA9_DN.V1	1 (0.9-1.11)	0.9777	0.95 (0.86-1.04)	0.2748	926
STK33	0.96 (0.85-1.08)	0.5012	0.92 (0.82-1.02)	0.1081	926
STK33_NOMO	0.97 (0.87-1.08)	0.6005	0.93 (0.84-1.03)	0.1466	926
STK33_SKM	1.01 (0.91-1.13)	0.8158	0.96 (0.87-1.06)	0.3856	926
KRAS.DF.V1	0.99 (0.89-1.1)	0.8243	0.94 (0.85-1.04)	0.208	926
TBK1.DF	0.97 (0.86-1.08)	0.5533	0.92 (0.84-1.02)	0.1187	926
JAK2_DN.V1	0.88 (0.81-0.96)	0.0045	0.86 (0.79-0.94)	0.0007	926
KRAS.300_UP.V1	0.83 (0.73-0.94)	0.0027	0.82 (0.73-0.92)	0.0005	926
KRAS.600_UP.V1	0.9 (0.78-1.03)	0.111	0.87 (0.78-0.98)	0.0249	926
KRAS.600.LUNG.BREAST_UP.V1	0.93 (0.81-1.07)	0.302	0.9 (0.8-1.01)	0.0735	926
KRAS.KIDNEY_UP.V1	0.89 (0.79-1.01)	0.0816	0.87 (0.78-0.97)	0.0137	926
KRAS.LUNG_UP.V1	0.97 (0.88-1.07)	0.5716	0.92 (0.84-1.02)	0.1215	926

LEF1_UP.V1	1 (0.89-1.12)	0.9628	0.94 (0.85-1.04)	0.2549	926

Table S6: Multivariate Cox-regression results of the 57 perturbation activity profiles in the ER+ endocrine treated samples from METABRIC, adjusted for either the Molecular Prognostic Index (MPI) of Teschendorff et al (Genome Biol. 2006) or the Genomic Grade Index (GGI) of Loi et al (JCO 2007). We give the hazard ratio (HR) + 95%CI, the log-rank test P-value, and the number of samples.
